# Extraction, Microencapsulation and Storage Stability of Bioactive Compounds from Avocado Peel (*Persea americana* Mill Var. Hass)

**DOI:** 10.3390/molecules31213821

**Published:** 2026-10-23

**Authors:** Ana Teresa López-Corona, Guillermo Osorio-Revilla, Ofelia Gabriela Meza-Márquez, Tzayhri Gallardo-Velázquez, Oswaldo Arturo Ramos-Monroy

**Affiliations:** 1 Departamento de Ingeniería Bioquímica, Escuela Nacional de Ciencias Biológicas-Zacatenco, Instituto Politécnico Nacional, Av. Wilfrido Massieu S/N, Esq. Cda. Miguel Stampa, Col. Unidad Profesional Adolfo López Mateos, Zacatenco, Alcaldía Gustavo A. Madero, Ciudad de Mexico 07738, Mexico; alopezc1505@alumno.ipn.mx (A.T.L.-C.); oramosm@ipn.mx (O.A.R.-M.); 2 Departamento de Biofísica, Escuela Nacional de Ciencias Biológicas-Santo Tomás, Instituto Politécnico Nacional, Prolongación de Carpio y Plan de Ayala S/N. Col. Santo Tomás, Alcaldía Miguel Hidalgo, Ciudad de Mexico 11340, Mexico; tgallardov@ipn.mx

**Keywords:** *Persea americana* Mill, avocado, bioactive compounds, spray drying, microencapsulation, storage

## Abstract

Avocado peel (*Persea americana* Mill var. Hass) is an important source of bioactive compounds with antioxidant activity; however, once extracted from the peel, these compounds are susceptible to deterioration caused by environmental factors such as light, oxygen, and temperature and therefore require protection. The objective of this study was to extract and microencapsulate the bioactive compounds from avocado peel and evaluate their stability during storage. Extraction conditions were optimized using a Box–Behnken design, resulting in 65 °C, 40%_V_ ethanol, and two sequential 60-min extractions. Microencapsulation was performed by spray drying using 10%_W_ and 20%_W_ gum arabic (GA) as wall material in the feed solution. The optimal drying conditions were 160/70 °C for 10%_W_ GA and 170/80 °C for 20%_W_ GA. Microcapsules containing 20%_W_ GA showed greater retention and stability of bioactive compounds than those containing 10%_W_ GA during 60 days of storage. At both GA concentrations, microcapsules stored at 4 °C showed greater stability than those stored at 30 °C. The results indicate that microencapsulated bioactive compounds extracted from avocado peel could be used in the food industry to develop functional foods or nutraceuticals.

## 1. Introduction

In recent years, interest in the development of functional foods obtained from natural sources has increased because, in addition to providing nutritional value, they offer additional health benefits [1,2].

Among the compounds of greatest interest are plant-derived bioactive compounds, also known as phytochemicals (e.g., phenolic acids, terpenes, flavonoids, and tannins), which are secondary plant metabolites. These compounds exhibit antioxidant, antimicrobial, anti-inflammatory, and other biological activities of interest to the food and pharmaceutical industries [3,4,5].

Fruits and vegetables are currently recognized as important sources of bioactive compounds; however, during their consumption and processing, only the pulp is generally consumed, generating by-products such as peels and seeds that are discarded even though they may contain higher amounts of bioactive compounds than the edible portion [6]. This is the case for avocado (*Persea americana* Mill var. Hass), one of the tropical fruits of greatest economic importance worldwide because of its extensive production [7].

Avocado is consumed mainly fresh; however, a substantial proportion of production is intended for industrial processing into oils, puree, guacamole, frozen cubes, and other derived products. Consequently, large amounts of agro-industrial waste are generated, mainly peel and seed, which account for approximately 30% of the total fruit weight. These by-products are commonly discarded, generating environmental problems and costs associated with their handling and final disposal [8,9].

Several studies have shown that avocado peel is an important source of phenolic compounds with notable biological activity, as it has a higher antioxidant content than the fruit pulp, even exceeding that of fresh blueberries [5,10,11].

Because of these characteristics, avocado peel has attracted interest as a raw material for obtaining bioactive compounds for potential incorporation into food products, since peel extracts have shown protective effects against oxidative stress and apoptosis in neuronal cell models [12,13].

Once extracted, these bioactive compounds are susceptible to degradation caused by several factors, including temperature, moisture, light, and oxygen [14]. These conditions may lead to losses in biological activity and antioxidant capacity; therefore, microencapsulation by spray drying has emerged as a strategy to protect bioactive compounds, because this technique allows the properties of the compounds to be preserved during processing and storage [15,16,17].

Although studies have focused on the extraction, identification, and quantification of bioactive compounds present in avocado peel, most have centered on evaluating their chemical composition and biological activity. Consequently, limited information is available on the protection of avocado peel bioactive compounds through spray-drying microencapsulation using gum arabic as the wall material.

Likewise, few studies have evaluated the stability of encapsulated bioactive compounds during storage, which is essential for determining their potential incorporation into functional foods.

Based on the above, the objective of this study was to extract bioactive compounds from avocado peel (*Persea americana* Mill var. Hass), microencapsulate them by spray drying using gum arabic as wall material, and evaluate the stability of the microencapsulated bioactive compounds during storage, analyzing the effect of wall material concentration on bioactive compounds retention, to assess its potential use in the development of functional foods and nutraceuticals.

## 2. Results and Discussion

### 2.1. Effect of Raw-Material Drying Temperature on the Concentration of Phenolic Compounds

Total phenolic compound (TPC) content was used as the response variable. The highest TPC concentration (43.31 mg GAE/g_dw_) was obtained in the sample dried at 40 °C for 24 h, representing a 5.64% increase relative to the control treatment dried at room temperature for 48 h (41.00 mg GAE/g_dw_).

In contrast, the sample dried at 50 °C for 24 h had a lower concentration (40.52 mg GAE/g_dw_) than the control, corresponding to 98.83% of the value observed in the latter. This result is consistent with the findings reported by Castillo-Zapata et al. [18], who indicated that the higher the drying temperature, the greater the decrease in phenolic compound content.

### 2.2. Extraction Under the Conditions Reported in Literature

The modifications made to the original method of Melgar et al. [19] (manual agitation every 10 min instead of constant agitation and a sample-to-solvent ratio of 1:10 instead of 1:25) resulted in the extraction of 91% of the phenolic compounds compared with the original method of Melgar et al. [19]. Therefore, for practical reasons, these modifications were adopted for the extraction of bioactive compounds from avocado peel in all subsequent stages of this study.

Because the phenolic compound content obtained using the modifications to the method of Melgar et al. [19], 21.90 mg GAE/g_dw_, was below the values reported by Salmerón-Ruíz [20], who reported 53.67 mg GAE/g_dw_, and Rodríguez-Carpena et al. [21], who reported 78.41 mg GAE/g_dw_, optimization of the extraction conditions was undertaken to maximize the recovery of phenolic compounds present in avocado peel.

### 2.3. Optimization of Extraction Conditions

Optimization of the extraction conditions using the Box–Behnken experimental design established the following optimal conditions: a temperature of 65 °C, a time of 60 min, and an ethanol concentration of 40%_V_ (Figure 1).

Under these conditions, the highest recovery of phenolic compounds and flavonoids from avocado peel was obtained, with a composite desirability of 0.9519. According to Derringer and Suich [22] and Aguirre-Solís [23], a desirability close to 1 indicates a high probability of achieving the optimal conditions for maximizing the extraction of phenolic compounds and flavonoids.

Figure 1 also presents plots of the effects of temperature, ethanol concentration, and time on the extraction of phenolic compounds and flavonoids. As temperature increases and ethanol concentration decreases from 80%_V_ to 40%_V_, the extraction of phenolic compounds and flavonoids increases. With respect to time, the response tends to remain nearly constant.

Once the optimal conditions had been determined through analysis of the experimental design, the values predicted by the model were compared with those obtained experimentally under these conditions. The results are presented in Table 1.

As shown in this table, the values predicted by the model and the experimental values did not differ significantly, indicating that the model generated using Minitab 22 software provided a good representation of the experimental results.

### 2.4. Extraction Kinetics

Once the optimal extraction conditions had been obtained and verified, extraction kinetics were evaluated to determine whether two 60 min extractions, as established by the method originally adopted from Melgar et al. [19], were actually necessary. The results showed that two consecutive 1 h extractions recovered more than 90% of these compounds, with cumulative recoveries of 91.30% for TPC and values above 90% for FL. In the third extraction, an additional 5.61% recovery was observed for phenolic compounds, whereas an additional 6.24% was obtained for flavonoids. Because of the low recovery percentages obtained in this final stage, two consecutive 1 h extractions were selected, since they were sufficient to achieve efficient recovery of phenolic compounds and flavonoids (>90%), consistent with Melgar et al. [19], who suggest this number of extractions.

### 2.5. Characterization of Bioactive Compounds Present in Avocado Peel Using Optimal Extraction Conditions

Table 2 presents the results corresponding to the characterization of bioactive compounds present in Hass avocado peel obtained under optimal extraction conditions (65 °C, 60 min, and 40%_V_ ethanol with two sequential extractions).

TPC results (Table 2) are comparable to those reported in the literature. Tremocoldi et al. [24] reported 63.5 ± 7.2 mg GAE/g of freeze-dried peel; Salmerón-Ruíz [20] reported 53.67 mg GAE/g_dw_; and Rodríguez-Carpena et al. [21] reported 78.41 mg GAE/g_dw_. These variations may be attributed to differences in extraction conditions, the type of solvent used, and sample pretreatment, such as freeze-drying.

For flavonoids, direct comparisons with other studies were not possible because those studies used quercetin (QE), rather than catechin (CE), as the reference compound.

The content of condensed and hydrolyzable tannins obtained in this study (31.81 ± 1.69 mg CE/g_dw_, 13.47 ± 0.93 mg TA/g_dw_) was higher than that reported by Salmerón-Ruíz [20], who reported values of 1.63 ± 0.01 mg CE/g_dw_ for condensed tannins and 5.79 ± 0.05 mg GAE/g_dw_ for hydrolyzable tannins. These differences could be attributed to several factors, including soil nutrient availability and fruit maturity [25].

It has been reported that lower nitrogen availability favors the accumulation of condensed tannins, whereas hydrolyzable tannins tend to occur at higher concentrations when plants reach advanced stages of maturation or are subjected to stress conditions, acting as defense mechanisms [26,27]. Likewise, changes in tannin structure occur during ripening, with an increase in the degree of polymerization of condensed tannins as this process progresses [28].

Regarding antioxidant capacity, the values obtained (Table 2) using DPPH (433.77 ± 20.86 µmol TE/g_dw_) and ABTS (503.67 ± 13.21 µmol TE/g_dw_) were within the ranges reported in other studies. For ABTS, Kosińska et al. [10] reported lower values (161 µmol TE/g_dw_), whereas Tremocoldi et al. [24] reported higher values of 791.5 ± 35.9 µmol TE/g_dw_. For DPPH, the value obtained in the present study was higher than that reported by Tremocoldi et al. [24], who obtained 310 ± 36.9 µmol TE/g_dw_. These variations may be associated with factors such as fruit origin, growing conditions, maturity, storage, and the extraction conditions used [29].

### 2.6. Determination of Bioactive Compounds and Antioxidant Capacity of Concentrated Avocado Peel Extract

Because the extract contained 40%_V_ ethanol, this solvent had to be removed by rotary evaporation. This step is essential to prevent the precipitation of the wall material during the preparation of the feed solution [30].

Table 3 presents the results obtained for the concentrated and unconcentrated extracts under the optimal extraction conditions, showing an increase in bioactive compound content due to ethanol evaporation.

The concentration of total phenolic compounds increased by 70% after the concentration process. Similarly, flavonoids increased by 89.13%, whereas hydrolyzable and condensed tannins increased by only 53.08% and 63.42%, respectively.

Likewise, antioxidant capacity increased by 50.22% and 66.02%, as determined by the DPPH and ABTS methods, respectively, which indicates that the antioxidant capacity determined by both methods is not directly related to phenol and flavonoid content, but probably to other compounds present in the extract, not determined in this work, and to CT and HT content, which showed similar percentage increment.

### 2.7. Optimization of the Spray-Drying Microencapsulation Process

Retention efficiency (%RE) and the content of bioactive compounds were determined in the microcapsules obtained under the different spray-drying conditions and wall-material concentrations (10%_w_ and 20% _w_ gum arabic).

Figure 2 shows the %RE values and concentrations of total phenolic compounds (TPC), flavonoids (FL), condensed tannins (CT), and antioxidant capacity (DPPH and ABTS; see the Abbreviations section). It should be noted that results for hydrolyzable tannins (HT) are not presented because they were not detectable in the samples analyzed after drying. The possible reason for this could be thermal degradation during the drying process plus the needed dilution of the sample to precipitate the wall material in microcapsules before analysis, which could make them undetectable by the analytical method used. It has been reported that HT are more sensitive to thermal degradation than CT [31,32,33]. Carsote et al. [31] and Miranda et al. [32] mention that due to the lower stability of chemical bonds in HT, they can be fragmented at lower temperatures than CT; therefore, CT are thermally more stable than HT. Pizzi et al. [33] indicate that in raw extracts containing carbohydrates, HT degradation temperature starts at approximately 150 °C. Since the inlet drying air temperature used in this work was 160–180 °C, this could cause degradation of the HT in microcapsules to a level that made them undetectable by the analytical method used.

As shown in Figure 2, in general, formulations prepared with 20%_W_ gum arabic exhibited higher retention percentages for all compounds evaluated, whereas those formulated with 10%_W_ showed higher concentrations of bioactive compounds, which is attributed to the smaller amount of wall material added.

The higher retention percentages of bioactive compounds observed in the microcapsules prepared with 20%_W_ GA are due to the protective effect of a greater proportion of gum arabic during spray drying, favoring the formation of a more stable encapsulating barrier that limits exposure of the bioactive compounds to the thermal conditions of the process.

Considering all treatments, Figure 2 shows that, overall, drying conditions had little effect on the retention of bioactive compounds, although significant differences were observed under some drying conditions for specific bioactive compounds.

In some cases, retention values slightly higher than 100% were observed in some formulations, especially in those capsules obtained with 20%_W_ GA. This could be due to the high inlet air temperatures used during the drying process, which could cause some phenolic compounds to be liberated from their conjugated form, increasing their reactivity to the Folin–Ciocalteu reagent. Similar results have been reported by Días et al. [34] and Toledo-Madrid et al. [35].

### 2.8. Optimal Spray-Drying Conditions for Avocado Peel Microcapsules

Figure 3 shows the optimal spray-drying conditions and the composite desirability plots obtained for formulations containing 10%_W_ (A) and 20%_W_ (B) gum arabic as the wall material.

In both cases, the optimization aimed to maximize antioxidant capacity and the content of phenolic compounds retained in Hass avocado peel microcapsules.

For the formulation containing 10%_W_ GA (Figure 3A), the software predicted an inlet temperature of 160 °C and an outlet temperature of 70 °C as the optimal conditions, reaching a composite desirability of 0.8292. In contrast, for the formulation containing 20%_W_ wall material (Figure 3B), the optimal conditions were an inlet temperature of 170 °C and an outlet temperature of 80 °C, with a composite desirability of 0.8095.

The differences in drying conditions between the two treatments may be attributed to the concentration of the wall material because a higher gum arabic content favors the formation of a more resistant wall during drying, allowing operation at slightly higher temperatures without compromising the stability of the bioactive compounds.

Table 4 shows the results predicted by the model and the values obtained experimentally under optimal drying conditions: 160/70 °C for 10%_W_ GA and 170/80 °C for 20%_W_ GA. As shown in this table, the predicted values were similar to those obtained experimentally, with no significant differences between them, indicating that the model was suitable for predicting optimal spray-drying conditions.

### 2.9. Characterization of Microcapsules Under Optimal Drying Conditions

#### 2.9.1. Particle Size and Particle-Size Distribution

The particle-size distribution of the microcapsules obtained is shown in Figure 4. Increasing the gum arabic concentration from 10%_W_ to 20%_W_ shifted the distribution toward larger sizes and increased its breadth, indicating greater dispersion in particle size, which can also be observed in Figure 5a–d.

Sauter diameter D [2,3] increased from 11.11 µm for 10%_W_ GA to 14.96 µm for 20%_W_ GA, whereas the equivalent sphere diameter D [3,4] increased from 13.56 to 18.35 µm, respectively. This behavior may be attributed to the increase in density and viscosity of the feed solution as the wall-material concentration increased [17]. Increased viscosity and density of the solution favor the formation of larger droplets during atomization, resulting in larger particles after drying [36,37].

#### 2.9.2. Microcapsule Morphology

Figure 5 presents images of avocado extract microcapsules obtained by spray drying using scanning electron microscopy (SEM).

In the micrographs corresponding to the formulation containing 10%_W_ gum arabic (Figure 5a,b), particles with surface deformations, such as wrinkles and shrunken surfaces, were observed, but with no evidence of fractures. These characteristics may be attributed to the drying conditions, since low or intermediate temperatures may lead to the formation of collapsed particles [38]. This phenomenon occurs when water diffuses from the interior of the particle through a wall that is more permeable to water vapor, allowing more time for the structures to deform, shrink, and ultimately collapse during the drying process [39].

In contrast, the micrographs corresponding to the formulation containing 20%_W_ GA (Figure 5c,d) show a predominance of larger particles than those obtained with 10%_W_ GA, consistent with the results in Figure 4. These particles also exhibited surface deformations, such as wrinkles and shrunken surfaces, but they were less pronounced than in microcapsules containing 10%_W_ GA. This morphology is similar to that reported by other authors who microencapsulated bioactive compounds extracted from different fruits and vegetable residues [30,40,41]. The increase in particle size with wall material concentration is related to the increase in both the concentration of wall material and the viscosity of the solution fed to the dryer, which favors the formation of larger particles with greater structural stability during drying and a surface less permeable to water vapor, reducing wall collapse and producing more well-defined microcapsules [17,42,43].

#### 2.9.3. Moisture Content and Water Activity (wa)

The moisture content of avocado peel extract microcapsules containing 10%_W_ and 20%_W_ GA ranged from 5.13 ± 0.305 to 5.53 ± 0.568%, values within the range reported for powders obtained by spray drying (1.50–9.55%) [44].

Water activity was low in both formulations, with values of 0.23 ± 0.009 for microcapsules prepared with 10%_W_ gum arabic and 0.17 ± 0.003 for those prepared with 20%_W_ gum arabic. These values are below the water activity of 0.3 reported for powders obtained by spray drying [45], which is considered favorable for maintaining product stability during storage [44]. Likewise, water activity values below 0.5 represent unfavorable conditions for microbial growth and development, contributing to the microbiological stability of the product [46].

#### 2.9.4. Phenolic Compounds on the Surface of the Microcapsules

Using the technique applied, detectable results for surface phenolics were obtained only for avocado peel microcapsules containing 10%_W_ GA. This behavior is consistent with the findings reported by Karamanoglu et al. [47], who stated that surface phenolic content decreases as the concentration of wall material increases.

The microcapsules formulated with 10%_W_ GA had a TPC content of 0.376 mg GAE/g_dw_, corresponding to 1.39% of the phenolic compounds present in the microcapsules. These results indicate that the vast majority of the phenolic compounds retained in the microcapsules are protected within the encapsulating matrix.

Using Equation 2, the encapsulation efficiency (%E), relative to the phenolic compound content in the feed solution, was determined to be 83.28%.

For the microcapsules formulated with 20%_W_ GA, the encapsulation efficiency was equal to or very close to the retention efficiency, indicating that virtually all of the retained compounds were encapsulated within the matrix.

### 2.10. Stability of Bioactive Compounds in Avocado Peel Microcapsules During Storage

Figure 6a–f shows that microcapsules containing either 10%_W_ or 20%_W_ wall material and stored at 4 °C exhibited greater stability than those stored at 30 °C, demonstrating the effect of storage temperature on the bioactive compounds. A three-way ANOVA (gum arabic concentration, storage temperature, storage time) was performed; main effects and interaction effects on each bioactive compound and antioxidant capacity are in Figures S1–S5. Raw retention data at each sampling time-point are in Table S1.

Microcapsules containing 10%_W_ GA stored at 30 °C showed the greatest losses of bioactive compounds. TPC showed losses of 6.34% at 4 °C and 7.72% at 30 °C, whereas flavonoids decreased by 3.26% and 5.37%, respectively.

Regarding antioxidant capacity evaluated by the DPPH method, the microcapsules showed greater decreases than those observed for phenolic compounds and flavonoids, with losses of 6.10% at 4 °C and 8.99% at 30 °C. In contrast, condensed tannins and antioxidant capacity measured by the ABTS assay showed smaller variations, with losses below 4.5% at both temperatures.

Microcapsules with 20%_W_ gum arabic concentration enhanced the protection of bioactive compounds, reducing degradation at both storage temperatures. At 30 °C, compound losses increased, especially for phenolic compounds (4.46%); however, these losses were lower than those observed with 10%_W_ GA.

The results obtained are consistent with previous studies reporting greater preservation of compounds in microencapsulated systems stored under refrigeration and formulated with different concentrations of gum arabic [17,48].

Finally, the results obtained during storage showed that 20%_W_ GA at 4 °C was the most suitable condition for preserving bioactive compounds and antioxidant capacity in avocado peel microcapsules. However, in addition to retention percentage, the final concentration of bioactive compounds is important to consider because a higher wall-material content improves stability but reduces the concentration of the encapsulated bioactive compounds.

## 3. Materials and Methods

### 3.1. Reagents and Wall Material

Gallic acid, Trolox (6-hydroxy-2,3,7,8-tetramethylchroman-2-carboxylic acid), aluminum chloride, potassium iodate, vanillin, tannic acid, 2,2′-azino-bis (3-ethylbenzothiazoline-6-sulfonic acid) (ABTS), and 2,2-diphenyl-1-picrylhydrazyl (DPPH) were purchased from Sigma-Aldrich (St. Louis, MO, USA). The Folin–Ciocalteu reagent was obtained from Hycel Reactivos Químicos (Mexico City, Mexico). Sodium carbonate, sodium nitrite, ethanol, methanol, and hexane were obtained from Meyer (Mexico City, Mexico). Hydrochloric acid and sodium hydroxide were obtained from Materiales y Abastos Especializados (Zapopan, Jalisco, Mexico). Gum arabic was purchased from Reasol (Mexico City, Mexico).

### 3.2. Avocado Samples

Avocado fruits (*Persea americana* Mill var. Hass) were obtained from different retail sources located in Ecatepec de Morelos, State of Mexico, including the Central de Abastos and supermarkets. In addition, avocado peels were collected from food preparation establishments to obtain a representative sample of this waste. Avocado peels from the different sources were analyzed for phenolic compounds using the methodology proposed by Melgar et al. [19]. The variation was from 15 to 25 mg GAE/g_dw_ depending on the source. Since most of the samples showed phenolic compound concentrations between 20 and 25 mg GAE/g_dw,_ this range was adopted. Samples that complied with this criterion were mixed together to obtain a representative sample for bioactive compounds extraction.

### 3.3. Sample Preparation

Avocado peels were selected, cleaned, and washed with abundant water. Excess moisture was removed with absorbent paper, and the peels were subsequently stored in airtight bags at −20 °C until use.

Before processing, the samples were thawed at room temperature. Thawed peels were cut into approximately 1 × 1 cm pieces and subjected to three drying treatments to evaluate the effect of raw-material drying on the extraction of bioactive compounds: (a) room temperature (27 °C ± 2 °C) for 48 h; (b) 40 °C in an oven (JEIO.TECH, OV-12, Waltham, MA, USA) for 24 h; and (c) 50 °C in an oven (JEIO.TECH, OV-12, Waltham, MA, USA) for 24 h.

The dried samples were ground using a food processor (Magic Bullet, MB1001, Los Angeles, CA, USA), and the resulting powder was sieved through a No. 20 mesh (850 µm). Finally, the samples were collected, labeled, and stored in airtight bags at −20 °C until use.

### 3.4. Extraction of Bioactive Compounds

Avocado peel extracts were obtained using the methodology proposed by Melgar et al. [19] with a ratio of 1 g of dry sample to 25 mL of ethanol (80%_V_), two sequential 1 h extractions at 25 °C, and constant agitation (150 rpm). These conditions were modified by using a sample-to-solvent ratio of 1:10, and constant agitation was replaced by manual agitation every 10 min during the extraction process.

Each extraction lasted 60 min, after which the samples were centrifuged at 3500 rpm for 15 min. The recovered supernatant was retained, and the solid residue was subjected to a second extraction under the same conditions. The supernatants obtained from both extractions were then combined and centrifuged again at 3500 rpm for 15 min. Finally, the extract was stored under refrigeration in amber glass bottles.

### 3.5. Optimization of Extraction Conditions

Extraction was optimized using a Box–Behnken experimental design. Due to the wide variability of extraction conditions for avocado peel bioactive compounds reported in the literature, including ethanol concentration (80%_v_ to 40%_v_), temperature (25 °C to 65 °C) and extraction time (30 min–60 min) [19,24,49,50], the variables considered for the optimization at two different levels (low and high) were: temperature (25 °C and 65 °C), time (30 min and 60 min), and ethanol concentration (40%_V_ and 80%_V_).

The experimental design was performed using MINITAB 22 software with a 95% confidence level and five replicates at the center point, yielding an experimental matrix of 17 runs shown in Table 5. The response variables were total phenolic compounds and flavonoids.

Once the optimal extraction conditions were obtained, extraction kinetics were evaluated over five consecutive extractions to determine the number of sequential extractions required to recover at least 90% of phenolic compounds and flavonoids.

### 3.6. Analytical Methods

#### 3.6.1. Total Phenolic Compounds (TPC)

TPC was determined using the Folin–Ciocalteu method described by Singleton and Rossi [51]. Sample absorbance was read at 750 nm (Jenway 6305, Long Branch, NJ, USA). The standard curve was prepared with gallic acid (0.01–0.2 mg/mL). For the extract, results were expressed as mg gallic acid equivalents per gram of extract (mg GAE/g_ext_), and for the microcapsules as mg gallic acid equivalents per gram of dry weight (mg GAE/g_dw_).

#### 3.6.2. Determination of Flavonoids (FLs)

FLs were determined using the colorimetric method described by Zhishen et al. [52]. Sample absorbance was read at 510 nm (Jenway 6305, Long Branch, NJ, USA). The standard curve was prepared with catechin (0.03–0.3 mg/mL). For the extract, results were expressed as mg catechin equivalents per gram of extract (mg CE/g_ext_), and for the microcapsules as mg catechin equivalents per gram of dry weight (mg CE/g_dw_).

#### 3.6.3. Determination of Condensed Tannins (CTs)

CTs were determined using the method of Sun et al. [53]. Sample absorbance was read at 500 nm (Jenway 6305, Long Branch, NJ, USA). The standard curve was prepared with catechin (0.015–0.120 mg/mL). For the extract, results were expressed as mg catechin equivalents per gram of extract (mg CE/g_ext_), and for the microcapsules as mg catechin equivalents per gram of dry weight (mg CE/g_dw_).

#### 3.6.4. Determination of Hydrolyzable Tannins (HTs)

HTs were determined using the method proposed by Çam and Hışıl [54]. Sample absorbance was read at 550 nm (Jenway 6305, Long Branch, NJ, USA). The standard curve was prepared with tannic acid (0.25–2.0 mg/mL). For the extract, results were expressed as mg tannic acid equivalents per gram of extract (mg TA/g_ext_), and for the microcapsules as mg tannic acid equivalents per gram of dry weight (mg TA/g_dw_).

#### 3.6.5. Determination of Antioxidant Capacity (AC)

AC was determined using two free-radical inhibition methods (DPPH and ABTS). For the DPPH method, the methodology proposed by Brand-Williams et al. [55] was followed: sample absorbance was read at 515 nm (Jenway 6305, Long Branch, NJ, USA). The standard curve was prepared with Trolox, 4 mM (40–1200 µmol). For the ABTS method, the methodology proposed by Re et al. [56] was followed: sample absorbance was read at 734 nm (Jenway 6305, Long Branch, NJ, USA). The standard curve was prepared with Trolox, 7 mM (50–2000 µmol). For both methods, results were expressed as micromoles of Trolox equivalents per gram of extract (µmol TE/g_ext_) for the extract and as micromoles of Trolox equivalents per gram of dry weight (µmol TE/g_dw_) for the microcapsules.

### 3.7. Microencapsulation

The hydroalcoholic avocado peel extract was subjected to evaporation to remove ethanol because its presence causes precipitation of the wall material (gum arabic) upon incorporation. Evaporation was carried out in a rotary evaporator (BUCHI, R-300, Flawil, Switzerland) at 120 rpm, 40 °C, and 77 mBar.

A 30%_W_ gum arabic solution in distilled water (*w*/*w*) was subsequently prepared. The mixture was homogenized and allowed to stand for 12 h to remove air trapped in the solution. A mass balance was then performed using this solution to calculate the amounts of gum arabic and concentrated extract (resulting from ethanol evaporation) required to adjust the wall-material concentration to 10%_W_ and 20%_W_ in the feed solution intended for spray drying.

The microencapsulation process was carried out using a semi-pilot spray dryer (GEA-NIRO Atomizer, Mobile Minor, Copenhagen, Denmark), which was equipped with a two-fluid spraying nozzle and a cyclone separator to gather the powder. The air spraying pressure was 0.5 kgf/cm^2^. Microencapsulation was performed at different inlet and air temperatures according to the experimental design mentioned in the next section. The powders obtained (microcapsules) were stored at −20 °C until analysis.

#### 3.7.1. Optimization of Spray-Drying Operating Conditions

Optimization was carried out using a factorial experimental design 2^2^ with three replicates at the center point. The variables evaluated in this design were inlet temperature (160 °C and 180 °C) and outlet temperature (70 °C and 90 °C). These conditions were selected based on previous reports on spray drying microencapsulation of bioactive compounds from other agro-industrial waste [35,41,57].

This analysis was performed independently for the two wall-material concentrations (10%_W_ and 20%_W_). The response variables were the retention efficiency (%RE) of TPC, FL, CT, HT, and AC (DPPH, ABTS). %RE was calculated using Equation (1).

(1) $$\%\mathrm{RE}=\frac{\mathrm{TPC},\mathrm{FL},\mathrm{CT},\mathrm{HT or AC content in the microcapsules}/g_{\mathrm{dw}}}{\mathrm{TPC},\mathrm{FL},\mathrm{CT},\mathrm{HT},\mathrm{or AC content in the feed solution}/g_{\mathrm{dw}}}\times 100$$

#### 3.7.2. Characterization of Microcapsules Under Optimal Drying Conditions

Microcapsules were reconstituted in water to the same solids content as the feed solution. Subsequently, for the determination of TPC, FL, CT, HT, and AC, the wall material had to be removed by ethanol precipitation to prevent interference from gum arabic in the measurements [41].

To precipitate the wall material, 1 mL of either the feed solution or the reconstituted microcapsules, as appropriate, was mixed with 1 mL of distilled water. From this mixture, 1 mL was taken, and 1.5 mL of absolute ethanol was added. The mixture was centrifuged (DESEGO, mCV-8, Mexico City, Mexico) at 10,000 rpm for 15 min, and the liquid phase was separated for analysis. This wall material precipitation method was validated against the theoretical content of each bioactive compound in the solution to be dried, based on the bioactive compound content in the concentrated extract of Table 3 and the amount of this solution required to prepare the drying solution for each wall material concentration.

#### 3.7.3. Determination of Surface Phenolic Compounds and Encapsulation Efficiency

The content of surface phenolic compounds was used as an indicator of the bioactive compounds present on the surface of the microcapsules, following the methodology proposed by Robert et al. [58]. Two grams of microcapsules were weighed, and 2 mL of a methanol:ethanol solution (1:1) was added. The mixture was then vortexed (Cole-Parmer 4554-10, Vernon Hills, IL, USA) for 1 min and subsequently vacuum-filtered (Whatman No. 1). Finally, the filtrate was analyzed to determine TPC. The encapsulation efficiency was determined using Equation (2):

(2) $$\%=\frac{\mathrm{Compound content in the microcapsules}/g_{\mathrm{dw}}-\mathrm{Compound content on the microcapsule surface}/g_{\mathrm{dw}}}{\mathrm{Compound content in the feed solution}/g_{\mathrm{dw}}}\times 100$$

#### 3.7.4. Moisture Content and Water Activity (aw)

Moisture content was determined using a moisture analyzer (OHAUS MB23, Mexico City, Mexico) at 100 °C for 15 min. Water activity (a_w_) was measured using an AquaLab Cx-3 (Decagon, Pullman, WA, USA).

#### 3.7.5. Particle Size and Particle-Size Distribution

The particle size and particle-size distribution of the microcapsules were determined using a Malvern laser-diffraction particle-size analyzer (IM 026 2006, Malvern, UK), with a 100 mm lens and hexane as the dispersing fluid.

#### 3.7.6. Microcapsule Morphology

Particle morphology was analyzed using a field-emission scanning electron microscope (FE-SEM, JEOL-JSM-7800F, Tokyo, Japan) operated at an accelerating voltage of 2 kV. Images were obtained at magnifications of 200× and 1000×. The analysis was performed at the Centro de Nanociencias y Micro y Nanotecnologías del Instituto Politécnico Nacional (CNMN-IPN).

#### 3.7.7. Stability of Bioactive Compounds of Microcapsules During Storage

Storage was carried out for 60 days at 4 °C and 30 °C. TPC, FL, CT, HT, and AC were analyzed every 15 days. Results were expressed as retention percentages based on the initial content (day 0) of bioactive compounds.

For storage, 3 g of microcapsules were weighed into amber bottles, which were labeled according to the analysis date (two bottles per date, independent replicates). The bottles were then closed and sealed with Parafilm to prevent moisture absorption. Analysis of each bioactive compound was performed in triplicate for each bottle every 15 days during storage, and the results were averaged.

### 3.8. Statistical Analysis

Results were evaluated by one-way analysis of variance (ANOVA), followed by comparison of means using Tukey’s test (*p* ≤ 0.05) with Minitab 22 software (Minitab, Inc., State College, PA, USA). Also, a three-way ANOVA (gum arabic concentration, storage temperature, and storage time) was performed to evaluate the main effects and interactions regarding each bioactive compound and antioxidant capacity during storage.

## 4. Conclusions

Optimal extraction conditions obtained with the Box–Behnken design were 65 °C, 40%_V_ ethanol, and 60 min, with two sequential extractions and agitation every 10 min, which allowed for recovery of more than 90% of the bioactive compounds present in avocado peel.

Regarding the optimization of spray drying, the optimal conditions were 160/70 °C (inlet/outlet temperature) for formulations containing 10%_W_ gum arabic and 170/80 °C for those containing 20%_W_. Microcapsules formulated with 20%_W_ gum arabic showed greater retention of phenolic compounds and antioxidant capacity, as well as greater stability during storage, particularly at 4 °C. However, increasing the wall-material concentration also reduced the concentration of phenolic compounds; therefore, selection of the most appropriate formulation will depend on the intended use of the potential functional food product. Thus, formulations with a lower wall-material concentration (10%_W_) may be preferable for products with a short shelf life under refrigeration because of their higher initial concentration of bioactive compounds, whereas formulations with a higher wall-material concentration (20%_W_) may be more suitable for products with a long shelf life at room temperature.

Finally, considering the retention of bioactive compounds as well as stability during storage, it can be concluded that microcapsules obtained by spray drying avocado peel extract could represent a promising alternative for use in the development of functional foods and nutraceuticals.

In future work, HPLC analysis will be performed on both the concentrated extract and microcapsules to identify the main bioactive compounds that were retained during the microencapsulation process, and not only as total phenolic compounds and flavonoids, as done in the present work. Also, although the total solids content in the concentrated extract is very low (about 1%_W_), efforts will be made to obtain the extract in powder form, probably by spray or freeze drying, to compare it with microcapsules during storage under the same conditions and in simulated digestion to evaluate the bioaccessibility of bioactive compounds in the extract and microcapsules.

## Figures and Tables

**Figure 1:**
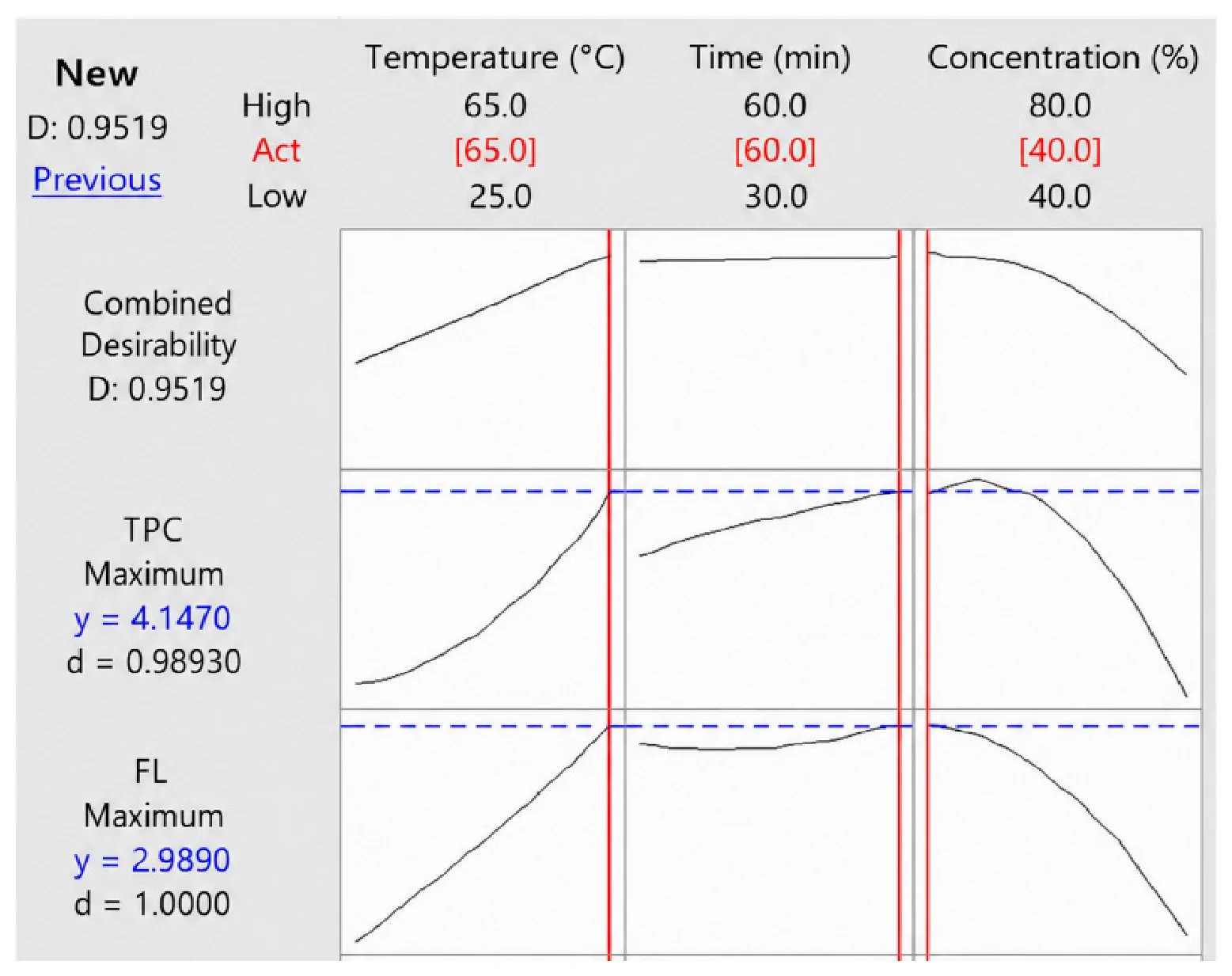
Optimal extraction conditions using the Box–Behnken design.

**Figure 2:**
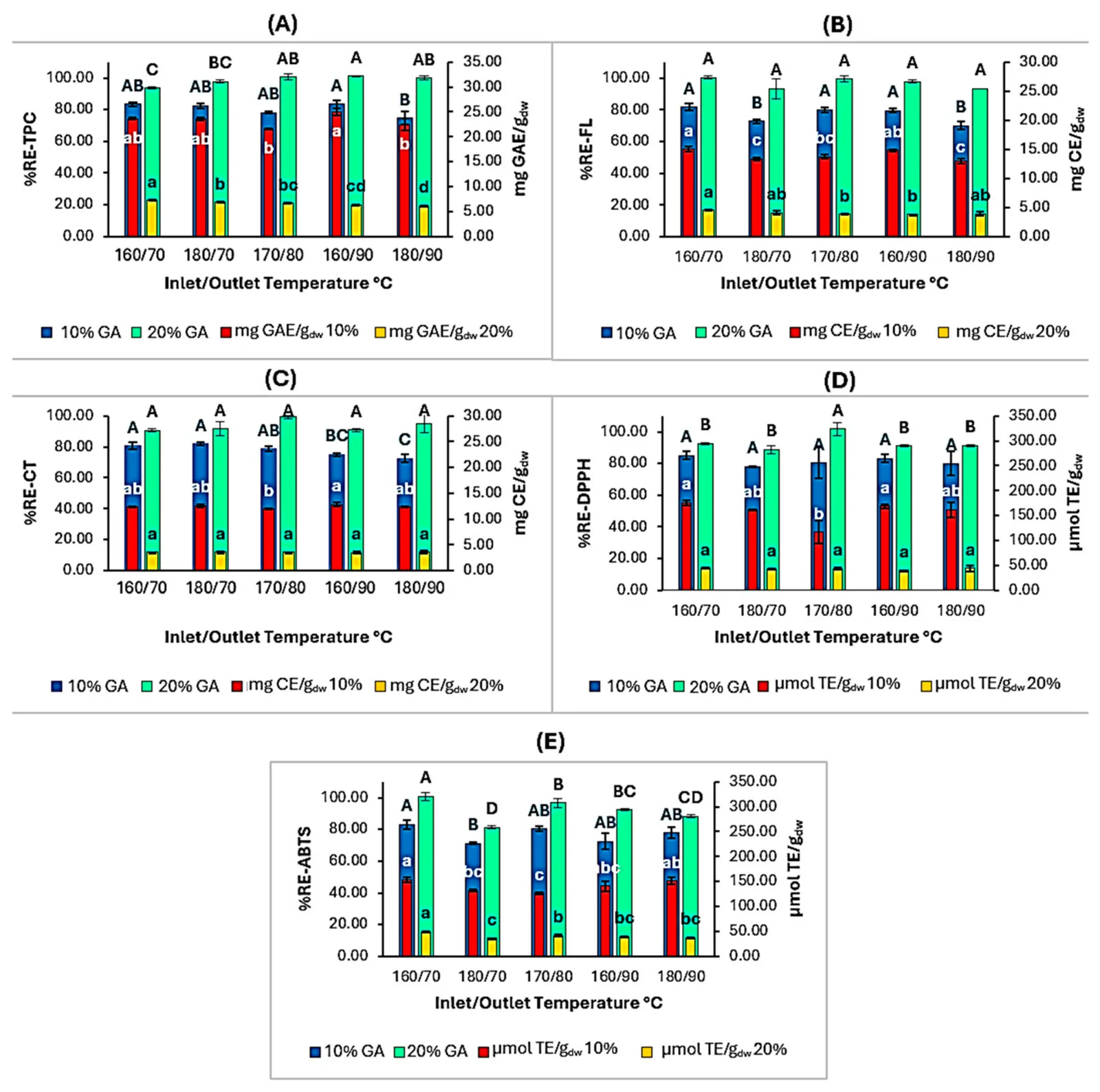
Retention efficiency (%RE) and bioactive compound content of microcapsules with 10%_W_ and 20%_W_ gum arabic. (**A**) Total phenolic compounds (TPC); (**B**) total flavonoids (FL); (**C**) condensed tannins (CT); antioxidant activity by: (**D**) DPPH; (**E**) ABTS. Data are expressed as mean ± standard deviation. Different letters indicate a significant difference (*p* ≤ 0.05) between drying conditions. Blue uppercase letters indicate a significant difference between formulations with 10%_W_ GA. Black uppercase letters compare formulations with 20%_W_ GA. White lowercase letters indicate a significant difference in bioactive compound content for formulations with 10%_W_ GA. Black lowercase letters compare formulations with 20%_W_ GA. See the Abbreviations section.

**Figure 3:**
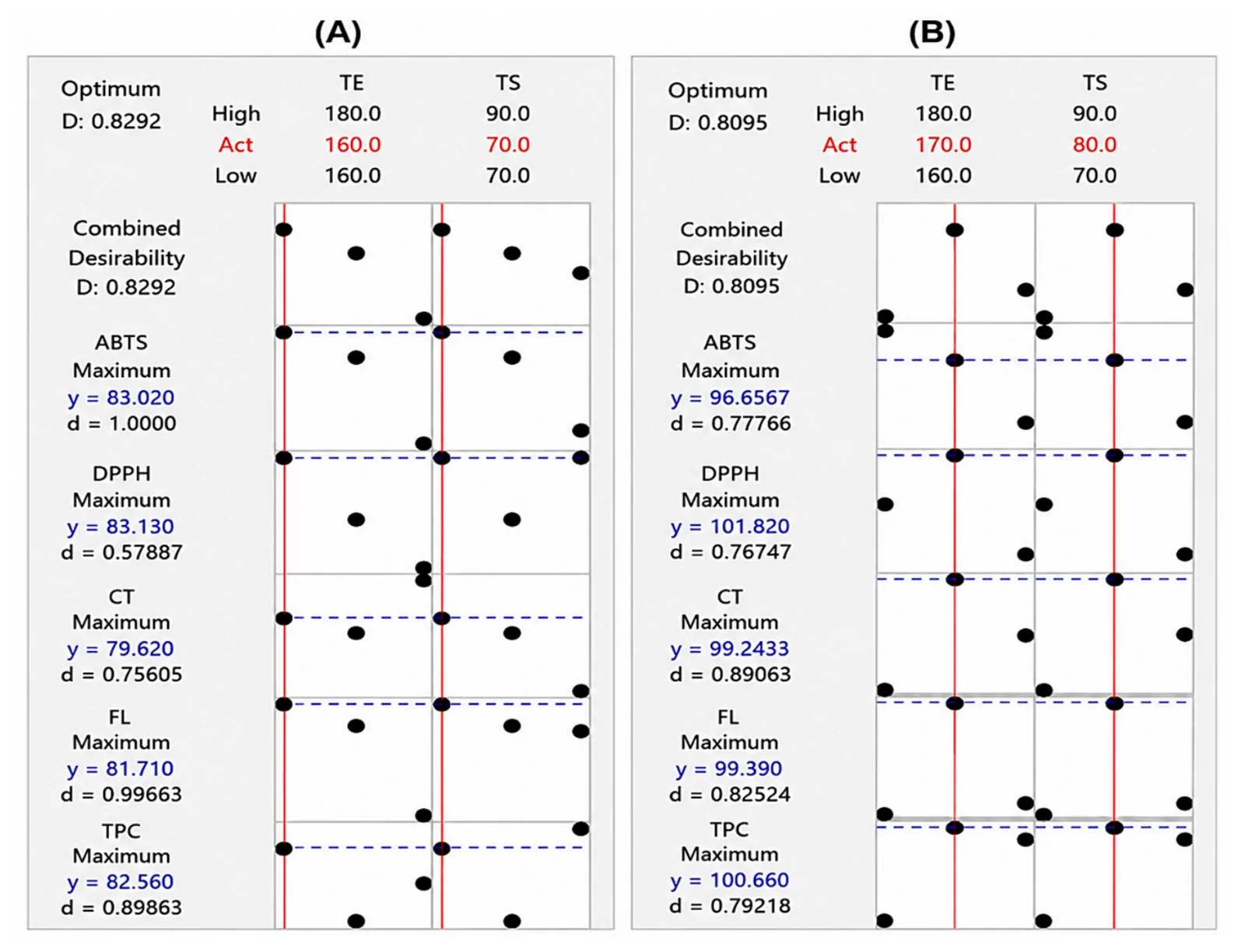
Optimal spray-drying conditions for microcapsules with 10%_W_ (**A**) and 20%_W_ (**B**) gum arabic.

**Figure 4:**
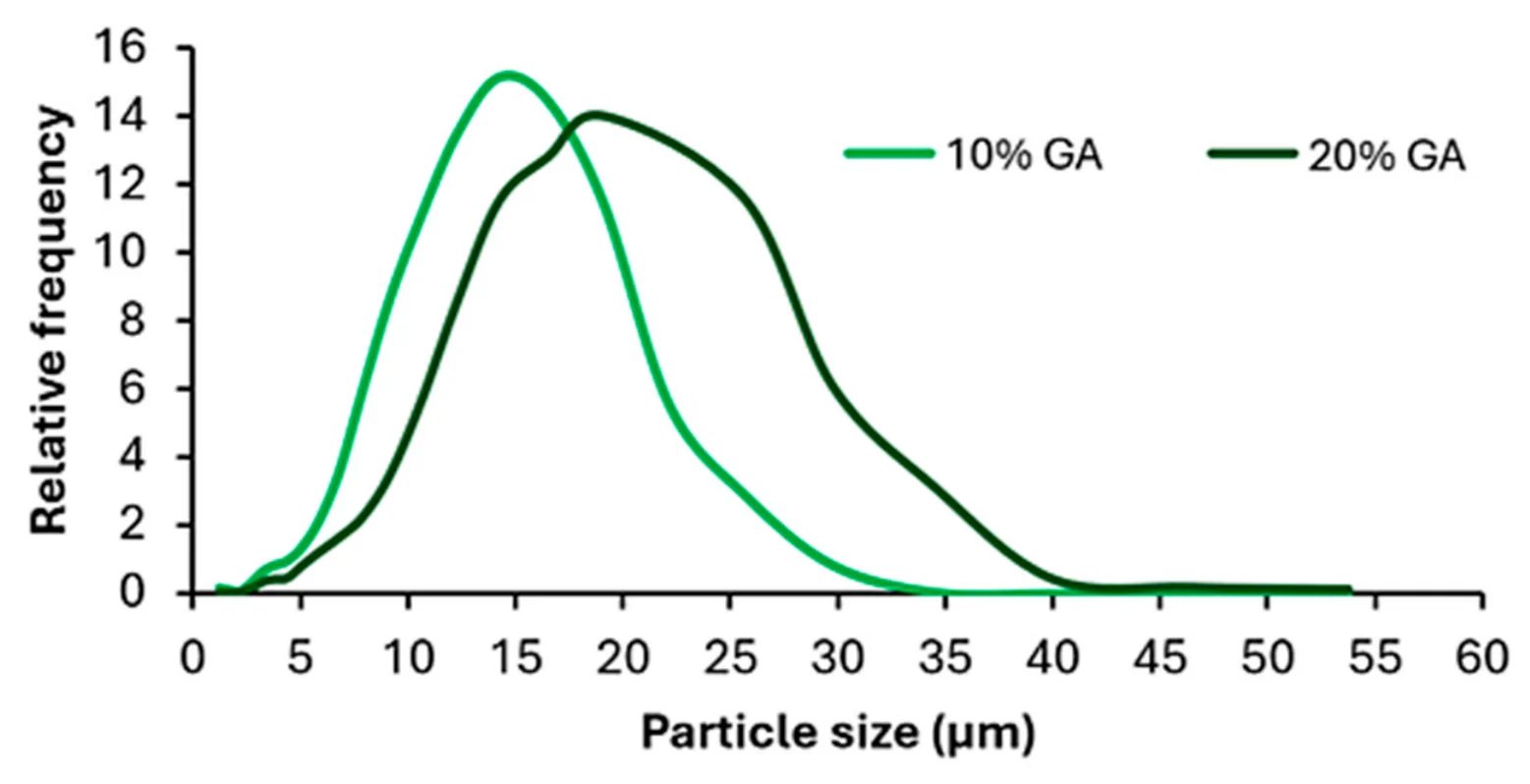
Particle-size distribution of microcapsules.

**Figure 5:**
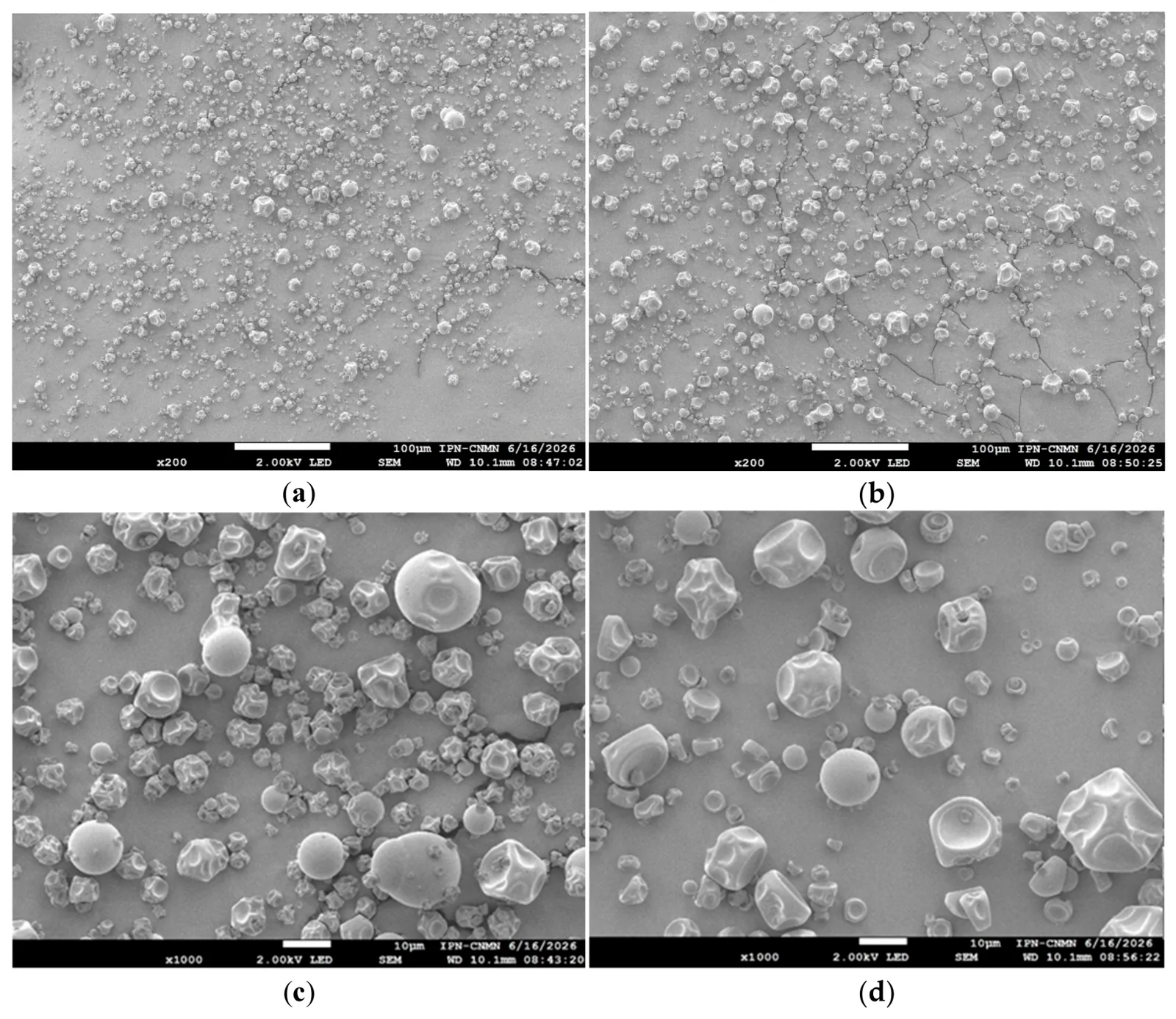
Scanning electron microscopy (SEM) micrographs of microcapsules. Microcapsules with 10%_W_ GA: (**a**) 200×; (**b**) 1000×. Microcapsules with 20%_W_ GA: (**c**) 200×; (**d**) 1000×.

**Figure 6:**
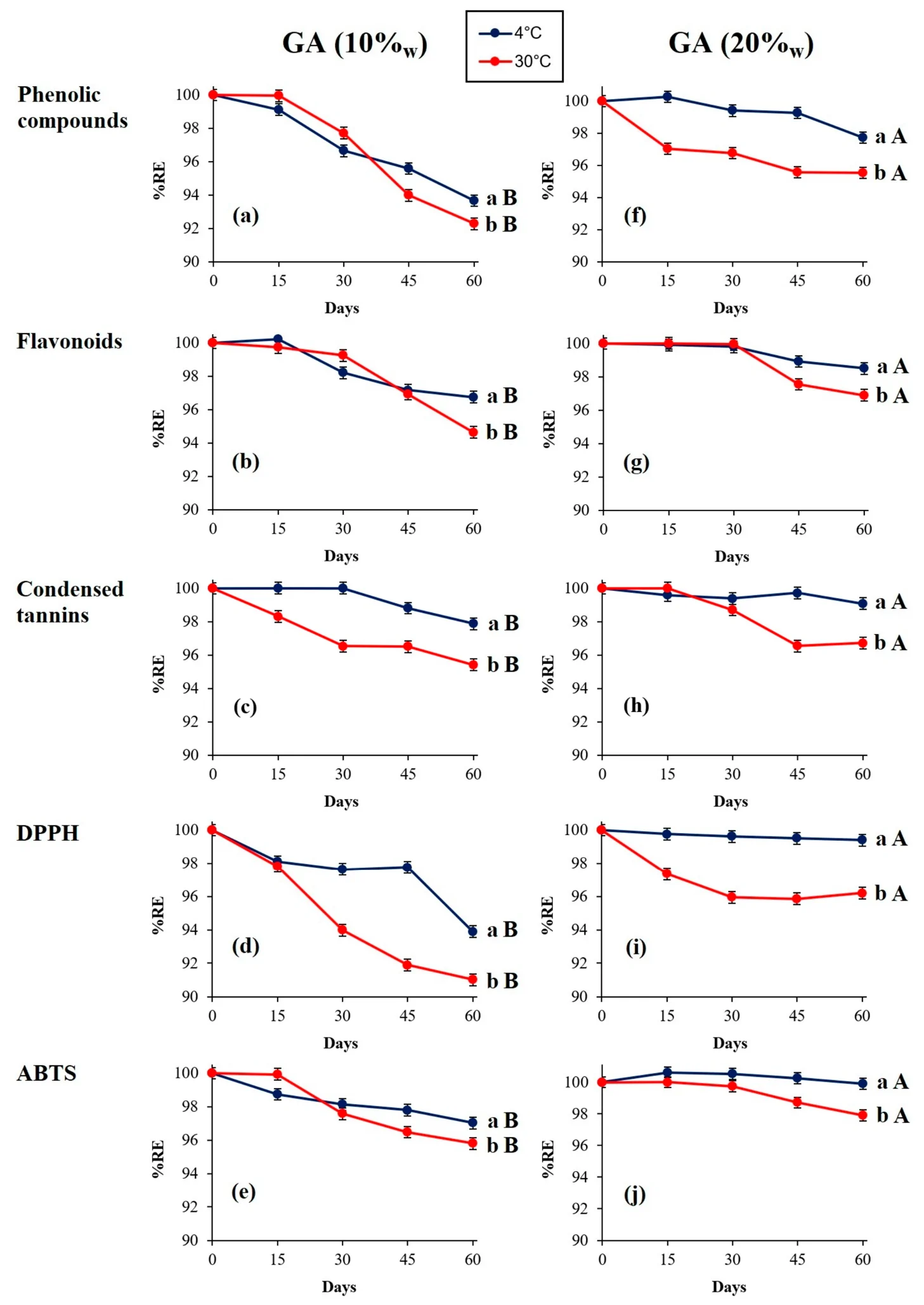
Retention efficiency (%RE) of bioactive compounds and antioxidant capacity of microcapsules with gum arabic (GA) at 10%_w_ (**a**–**e**) and 20%_w_ (**f**–**j**). Data are expressed as mean ± standard deviation. Means with different lowercase letters are significantly different (*p* ≤ 0.05) between temperatures for the same bioactive compound or antioxidant capacity at the same GA concentration. Means with different uppercase letters are significantly different (*p* ≤ 0.05) between GA concentrations for the same bioactive compound or antioxidant capacity at the same temperature.

**Table 1:** Comparison of experimental values with predicted values by the model of Hass avocado peel under optimal conditions.

Determination	Predicted Value	Experimental Value	*p*-Value
TPC (mg GAE/g_ext_)	4.15	4.29 ± 0.06	0.056
FL (mg CE/g_ext_)	2.99	2.67 ± 0.17	0.083

Values are expressed as mean ± standard deviation (*n* = 3). See the Abbreviations section.

**Table 2:** Bioactive compounds of Hass avocado peel under optimal extraction conditions.

Determination	Content
TPC (mg GAE/g_dw_)	61.27 ±1.53
FL (mg CE/g_dw_)	38.08 ± 0.79
CT (mg CE/g_dw_)	31.81 ±1.69
HT (mg TA/g_dw_)	13.47 ± 0.93
DPPH (µmol TE/g_dw_)	433.77 ± 20.86
ABTS (µmol TE/g_dw_)	503.67 ± 13.21

Values are expressed as mean ± standard deviation (*n* = 3). See the Abbreviations section.

**Table 3:** Bioactive compounds and antioxidant capacity of optimized (unconcentrated) and concentrated extract.

Determination	Optimized Extract	Concentrated Extract
TPC (mg GAE/g_ext_)	4.29 ± 0.06	7.30 ± 0.10
FL (mg CE/g_ext_)	2.67 ± 0.07	5.05 ± 0.35
CT (mg CE/g_ext_)	2.57 ± 0.23	4.20 ± 0.37
HT (mg TA/g_ext_)	3.73 ± 0.30	5.71 ± 0.0
DPPH (µmol TE/g_ext_)	31.72 ± 1.76	47.65 ± 1.57
ABTS (µmol TE/g_ext_)	32.44 ± 1.03	53.86 ± 0.87

Values are expressed as mean ± standard deviation (*n* = 3). See the Abbreviations section.

**Table 4:** Comparison between predicted and experimental values for the retention of bioactive compounds and antioxidant capacity of microcapsules (10%_W_ and 20%_W_ of gum arabic, GA) obtained under optimal spray-drying conditions.

	Determination	Predicted %RE	Experimental %RE	Percentage Error (%)	*p*-Value
10%_W_ GA	TPC	82.56	84.43 ± 1.05	2.21	0.375
	FL	81.71	83.07 ± 0.88	1.64	0.272
	CT	79.62	80.35 ± 1.23	0.91	0.412
	DPPH	83.13	81.37 ± 1.13	2.16	0.114
	ABTS	83.02	81.51 ± 2.25	1.85	0.365
20%_W_ GA	TPC	100.66	101.67 ± 1.21	1.00	0.301
	FL	99.39	99.10 ± 0.55	0.29	0.458
	CT	99.24	98.17 ± 0.75	1.08	0.132
	DPPH	101.82	99.46 ± 3.11	2.32	0.319
	ABTS	96.65	96.22 ± 2.75	0.44	0.081

Values are expressed as mean ± standard deviation (*n* = 3). See the Abbreviations section.

**Table 5:** Box–Behnken experimental matrix.

Experimental Run	Temperature (°C)	Time (min)	Concentration (%)
1	25	45	80
2	45	60	80
3	45	45	60
4	45	30	40
5	65	60	60
6	65	45	40
7	45	45	60
8	25	60	60
9	25	30	60
10	45	60	40
11	45	30	80
12	45	45	60
13	45	45	60
14	45	45	60
15	65	45	80
16	25	45	40
17	65	30	60

## Data Availability

The original contributions presented in the study are included in the article; further inquiries can be directed to the corresponding author.

## Supplementary Materials

The following supporting information can be downloaded at: https://www.mdpi.com/article/10.3390/molecules31213821/s1, Figure S1: Main effects plot for phenolic compounds. Figure S2: Main effects plot for flavonoids. Figure S3: Main effects plot for tannins. Figure S4: Main effects plot for DPPH. Figure S5: Main effects plot for ABTS. Table S1: Raw storage data.

## Abbreviations

The following abbreviations are used in this manuscript:

GA	Gum arabic
ABTS	2,2′-Azino-bis (3-ethylbenzothiazoline-6-sulfonic acid
DPPH	2,2-Diphenyl-1-picrylhydrazyl
TPC	Total phenolic compounds
FL	Flavonoid
CT	Condensed tannin
HT	Hydrolyzable tannin
AC	Antioxidant capacity
%RE	Retention efficiency
GAE	Gallic acid equivalents
CE	Catechin equivalents
TA	Tannic acid
µmol TE	Micromol of Trolox equivalents

## References

[1] Jadhav H.B. (2024). Functional Triacylglycerols from Microalgae and Their Use in the Formulation of Functional Foods—Review. Food Chem. Adv..

[2] Mondal D., Awana M., Mandal S., Pandit K., Singh A., Syeunda C.O., Thandapilly S.J., Krishnan V. (2024). Functional Foods with a Tailored Glycemic Response Based on Food Matrix and Its Interactions: Can It Be a Reality?. Food Chem..

[3] Gasaly N., Riveros K., Gotteland M., Gasaly N., Riveros K., Gotteland M. (2020). Fitoquímicos: Una Nueva Clase de Prebióticos. Rev. Chil. Nutr..

[4] Urrialde R., Gómez-Cifuentes A., Pintos B., Gómez-Garay M.A., Cifuentes B. (2022). Compuestos Bioactivos de Origen Vegetal: Desarrollo de Nuevos Alimentos. Nutr. Hosp..

[5] Zaldivar-Ortega A.K., Barrera-Jiménez J.A., Cenobio-Galindo A.d.J., Pérez-Soto E., Franco-Fernández M.J., Campos-Montiel R.G. (2023). Potencial uso de la cáscara y semilla de aguacate como fuente de compuestos bioactivos con actividades funcionales para un desarrollo sustentable. Bol. Cienc. Agropecu. ICAP..

[6] Cid-Pérez T.S., Hernández-Carranza P., Ochoa-Velasco C.E., Ruiz-López I.I., Nevárez-Moorillón G.V., Ávila-Sosa R. (2021). Avocado seeds (*Persea americana* cv. Criollo sp.): Lipophilic compound profile and biological activities. Saudi J. Biol. Sci..

[7] Muhammad D.R.A., Ayouaz S., Rachmawati A.N., Madani K., Fibri D.L.N., Rafi M., Julianti E., Fahmy K. (2024). Advanced and Potential Methods for Extraction of Bioactive Compounds from Avocado Peel—A Review. Appl. Sci..

[8] Avila N.d.D., Tirado-Gallegos J.M., Rios-Velasco C., Esquivel G.L., Virgen M.O.E., Campos O.J.C. (2023). Propiedades composicionales, estructurales y fisicoquímicas de las semillas de aguacate y sus potenciales usos agroindustriales. Cienc. Tecnol. Agropecu..

[9] Salazar-López N.J., Domínguez-Avila J.A., Yahia E.M., Belmonte-Herrera B.H., Wall-Medrano A., Montalvo-González E., González-Aguilar G.A. (2020). Avocado Fruit and By-Products as Potential Sources of Bioactive Compounds. Food Res. Int..

[10] Kosińska A., Karamać M., Estrella I., Hernández T., Bartolomé B., Dykes G.A. (2012). Phenolic Compound Profiles and Antioxidant Capacity of *Persea americana* Mill. Peels and Seeds of Two Varieties. J. Agric. Food Chem..

[11] Velderrain-Rodríguez G.R., Quero J., Osada J., Martín-Belloso O., Rodríguez-Yoldi M.J. (2021). Phenolic-Rich Extracts from Avocado Fruit Residues as Functional Food Ingredients with Antioxidant and Antiproliferative Properties. Biomol..

[12] Nascimento A.P.S., Duarte M.E.M., Rocha A.P.T., Barros A.N. (2025). Valorization of Avocado (*Persea americana*) Peel and Seed: Functional Potential for Food and Health Applications. Antioxidants.

[13] Nyakang’i C.O., Ebere R., Marete E., Arimi J.M. (2023). Avocado production in Kenya in relation to the world, Avocado by-products (seeds and peels) functionality and utilization in food products. App. Food Res..

[14] Laureanti E.J.G., Paiva T.S., de Matos Jorge L.M., Jorge R.M.M. (2023). Microencapsulation of Bioactive Compound Extracts Using Maltodextrin and Gum Arabic by Spray and Freeze-Drying Techniques. Int. J. Biol. Macromol..

[15] Ortiz Sánchez C.A., Bonilla Zavaleta E., Urrea García G.R., Luna Solano G., Rascón Díaz M.P. (2021). Krill oil microencapsulation: Antioxidant activity, astaxanthin retention, encapsulation efficiency, fatty acids profile, in vitro bioaccessibility and storage stability. LWT.

[16] Zafar T., Sidhu J.S., Sinha N. (2011). Avocado: Production, Quality, and Major Processed Products. Handbook of Vegetables and Vegetable Processing.

[17] Quiroz Zarco J.E., Meza-Márquez O.G., Osorio-Revilla G., Gallardo-Velázquez T., Ramos-Monroy O.A. (2026). Stability of Phytochemical Compounds and Antioxidant Capacity of Garambullo (*Myrtillocactus geometrizans*) Extract Microencapsulated with Different Concentrations of Wall Material (Gum Arabic) During Storage and In Vitro Digestion. Foods.

[18] Castillo-Zapata K.C., Reyes-Diaz J.D., Cornelio-Santiago H.P., Espinoza-Espinoza L.A., Valdiviezo-Marcelo J., Ruiz-Flores L.A. (2024). Efecto del secado con aire caliente en el contenido de fenólicos totales y capacidad antioxidante de la cáscara de pitahaya roja (*Hylocereus guatemalensis*). Rev. Investig. Univ. Cordon Bleu.

[19] Melgar B., Dias M.I., Ciric A., Sokovic M., Garcia-Castello E.M., Rodriguez-Lopez A.D., Barros L., Ferreira I.C.R.F. (2018). Caracterización Bioactiva de Los Subproductos de *Persea americana* Mill.: Una Rica Fuente de Antioxidantes Inherentes. Ind. Crops Prod..

[20] Salmerón-Ruíz M. (2014). Fracción Indigestible, Bioaccesibilidad In Vitro y Actividad Antioxidante, de Compuestos Fenólicos de la Cáscara de Aguacate cv. ‘Hass’. Master’s Thesis.

[21] Rodríguez-Carpena J.-G., Morcuende D., Andrade M.-J., Kylli P., Estévez M. (2011). Avocado (*Persea americana* Mill.) Phenolics, in Vitro Antioxidant and Antimicrobial Activities, and Inhibition of Lipid and Protein Oxidation in Porcine Patties. J. Agric. Food Chem..

[22] Derringer G., Suich R. (1980). Simultaneous Optimization of Several Response Variables. J. Qual. Technol..

[23] Aguirre-Solís J.J., Portillo R.A.C., Hernández M.G.G., Hernández P.R.G., Ibarra-Mejía G., Anaya L.G.G. (2024). Rediseño de estación de trabajo mediante simulación humano digital y diseño de experimentos. Cult. Cient. Tecnol..

[24] Tremocoldi M.A., Rosalen P.L., Franchin M., Massarioli A.P., Denny C., Daiuto É.R., Paschoal J.A.R., Melo P.S., Alencar S.M. (2018). de Exploration of Avocado By-Products as Natural Sources of Bioactive Compounds. PLoS ONE.

[25] Lyu X., Agar O.T., Barrow C.J., Dunshea F.R., Suleria H.A.R. (2023). Phenolic Compounds Profiling and Their Antioxidant Capacity in the Peel, Pulp, and Seed of Australian Grown Avocado. Antioxidants.

[26] Olivas-Aguirre F.J., Wall-Medrano A., González-Aguilar G.A., López-Díaz J.A., Álvarez-Parrilla E., Rosa L.A.d.l., Ramos-Jimenez A. (2015). Taninos Hidrolizables: Bioquímica, Aspectos Nutricionales y Analíticos y Efectos En La Salud. Nutr. Hosp..

[27] Vaca-Sánchez M.S., González-Rodríguez A., Maldonado-López Y., Fernandes W.G., Cuevas-Reyes P. (2016). Importancia de los taninos en especies del género *Quercus* como metabolitos secundarios asociados a defensa contra insectos herbívoros. Biológicas.

[28] Vázquez-Flores A.A., Álvarez-Parrilla E., López-Díaz J.A., Wall-Medrano A., Rosa L.A.D. (2012). la Taninos hidrolizables y condensados: Naturaleza química, ventajas y desventajas de su consumo: Hydrolyzable and condensed tannins: Chemistry, advantages and disadvantages of their intake. Tecnocienc. Chihuah..

[29] Staveckienė J., Kulaitienė J., Levickienė D., Vaitkevičienė N., Vaštakaitė-Kairienė V. (2023). The Effect of Ripening Stages on the Accumulation of Polyphenols and Antioxidant Activity of the Fruit Extracts of Solanum Species. Plants.

[30] Roa-Tort A., Meza-Márquez O.G., Osorio-Revilla G., Gallardo-Velázquez T., Ramos-Monroy O.A. (2024). Extraction and Microencapsulation of Phytochemical Compounds from Mango Peel (*Mangifera indica* L.) Var. “Kent” and Assessment of Bioaccessibility through In Vitro Digestion. Processes.

[31] Carsote C., Balzano A., Colombini M.P., Coniglio Moskovics R., Degano I., Herrera Diaz R., Karapanagiotis I., Kilic-Pekgözlü A., Lucejko J.J., Yuste Córdoba F.J. (2026). Analytical Approaches for the Identification and Characterization of Tannins in Natural Matrices. A Comprehensive Review. Crit. Rev. Anal. Chem..

[32] Miranda R., Sosa E., Bustos C., Navarrete J. (2019). Pyrolysis-GC/MS and TGA analysis of condensed tannins extracted from *Acacia mearnsii* bark. J. Anal. Appl. Pyrolysis..

[33] Pizzi A. (2009). Tannins: Major Sources, Properties and Applications. J. Wood Chem. Technol..

[34] Díaz Y.L., Torres L.S., Serna J.A., Sotelo L.L. (2017). Efecto de la encapsulación en secado por atomización de biocomponentes de pitahaya amarilla con interés funcional. Inf. Tecnol..

[35] Toledo-Madrid K., Gallardo-Velázquez T., Osorio-Revilla G. (2018). Microencapsulation of Purple Cactus Pear Fruit (*Opuntia ficus indica*) Extract by the Combined Method W/O/W Double Emulsion-Spray Drying and Conventional Spray Drying: A Comparative Study. Processes.

[36] Esquivel-González B.E., Ochoa-Martínez L.A., Rutiaga-Quiñones O.M. (2015). Microencapsulación mediante secado por aspersión de compuestos bioactivos. Rev. Iber. Tecnol. Postcosecha.

[37] Ruíz-Aguilar I., Meza-Márquez O.G., Osorio-Revilla G., Gallardo-Velázquez T., Ramos-Monroy O.A. (2024). Phytochemical Compounds, and Antioxidant, Anti-Hyperglycemic, and Anti-Inflammatory Activity of Microencapsulated Garambullo (*Myrtillocactus geometrizans*) Extract During In Vitro Digestion and Storage. Processes.

[38] Jimenez-Gonzalez O., Luna-Guevara J.J., Ramírez-Rodrigues M.M., Luna-Vital D., Luna-Guevara M.L. (2022). Microencapsulation of *Renealmia alpinia* (Rottb.) Maas Pulp Pigment and Antioxidant Compounds by Spray-Drying and Its Incorporation in Yogurt. J. Food Sci. Technol..

[39] Rodsuwan U., Vatanyoopaisarn S., Thumthanaruk B., Thisayakorn K., Uttapap D., Zhong Q., Rungsardthong V. (2025). Physicochemical Properties of Spray-Dried Microcapsules Prepared with Gamma Oryzanol Pre-encapsulated in Zein Nanoparticles and Maltodextrin as a Drying Agent. Food Bioprocess Technol..

[40] Ramírez Damián M., Meza-Márquez O.G., Osorio-Revilla G., Gallardo-Velázquez T., Téllez-Medina D.I., Ramos-Monroy O.A. (2022). Microencapsulation of Red Banana Peel Extract and Bioaccessibility Assessment by In Vitro Digestion. Processes.

[41] Boyano-Orozco L., Gallardo-Velázquez T., Meza-Márquez O.G., Osorio-Revilla G. (2020). Microencapsulation of Rambutan Peel Extract by Spray Drying. Foods.

[42] Masters K. (1985). Spray Drying Handbook.

[43] Le P.-Q., Kim J.H., Park K., Lee B.-J. (2026). Design and Physicochemical Characterization of Spray-Dried Microcapsules Encapsulating o/w Emulsion-Based Drug Combinations. Int. J. Pharm..

[44] Sirichokworrakit S., Aukkanit N., Sangsuwon C. (2025). Optimización de Las Estrategias de Encapsulación Para Mejorar La Estabilidad Antioxidante de Los Extractos de *Wolffia globosa*: Un Estudio Comparativo de Maltodextrina, Goma Arábiga y Concentrado de Proteína de Suero. Appl. Food Res..

[45] Rezende Y.R.R.S., Nogueira J.P., Narain N. (2018). Microencapsulación de Extractos de Compuestos Bioactivos Obtenidos de Pulpa y Residuos de Acerola (*Malpighia emarginata* DC) Mediante Secado Por Aspersión y Liofilización: Caracterización Química, Morfológica y Quimiométrica. Food Chem..

[46] Fennema O.R., Fennema O. (2000). Agua y hielo. Química de los Alimentos.

[47] Karamanoglu A.A., Tarakçi Z., Karagol M.A. (2026). Encapsulation of Cherry Laurel (*Prunus laurocerasus*) Extracts via Spray and Freeze Drying: Effects on Bioactive Compounds, Volatile Profile, and Physicochemical Properties of the Powder. Food Sci. Nutr..

[48] Chen S., Fan L., Chen X., Guo Z., Zhang B. (2025). Microencapsulación Del Extracto de Orujo *de Lonicera Caerulea* Mediante Secado Por Aspersión: Caracterización y Estudios de Estabilidad. LWT.

[49] Araujo R.G., Rodríguez-Jasso R.M., Ruíz H.A., Govea-Salas M., Pintado M., Aguilar C.N. (2021). Recovery of Bioactive Components from Avocado Peels Using Microwave-Assisted Extraction. Food Bioprod. Process..

[50] Segovia F.J., Hidalgo G.I., Villasante J., Ramis X., Almajano M.P. (2018). Avocado Seed: A Comparative Study of Antioxidant Content and Capacity in Protecting Oil Models from Oxidation. Molecules.

[51] Singleton V., Rossi J.A. (1965). Colorimetry of total phenolics with phosphomolybdic-phosphotungstic and reagents. Am. J. Enol. Vitic..

[52] Jia Z., Tang M., Wu J. (1999). The Determination of Flavonoid Contents in Mulberry and Their Scavenging Effects on Superoxide Radicals. Food Chem..

[53] Sun B., Ricardo-da-Silva J.M., Spranger I. (1998). Critical Factors of Vanillin Assay for Catechins and Proanthocyanidins. J. Agric. Food Chem..

[54] Çam M., Hışıl Y. (2010). Pressurised Water Extraction of Polyphenols from Pomegranate Peels. Food Chem..

[55] Brand-Williams W., Cuvelier M.E., Berset C. (1995). Use of a Free Radical Method to Evaluate Antioxidant Activity. LWT-Food Sci. Technol..

[56] Re R., Pellegrini N., Proteggente A., Pannala A., Yang M., Rice-Evans C. (1999). Antioxidant Activity Applying an Improved ABTS Radical Cation Decolorization Assay. Free Radic. Biol. Med..

[57] Castro-Alatorre N.C., Gallardo-Velázquez T., Boyano-Orozco L.C., Téllez-Medina D.I., Meza-Márquez O.G., Osorio-Revilla G. (2021). Extraction and Microencapsulation of Bioactive Compounds from Muicle (*Justicia spicigera*) and Their Use in the Formulation of Functional Foods. Foods.

[58] Robert P., Gorena T., Romero N., Sepulveda E., Chavez J., Saenz C. (2010). Encapsulation of Polyphenols and Anthocyanins from Pomegranate (*Punica granatum*) by Spray Drying. Int. J. Food Sci. Technol..

