# Supplementary material for: Extraction, Microencapsulation and Storage Stability of Bioactive Compounds from Avocado Peel (Persea americana Mill Var. Hass)
Source: Molecules. 2026 Oct 23;31(21):3821. doi: 10.3390/molecules31213821 (PMC13765412; doi:10.3390/molecules31213821)
Supplement: Supplementary file 1 [file molecules-31-03821-s001.zip › molecules-4608092-Supplementary Material.pdf]

Figure S1. Main effects plot for phenolic compounds.

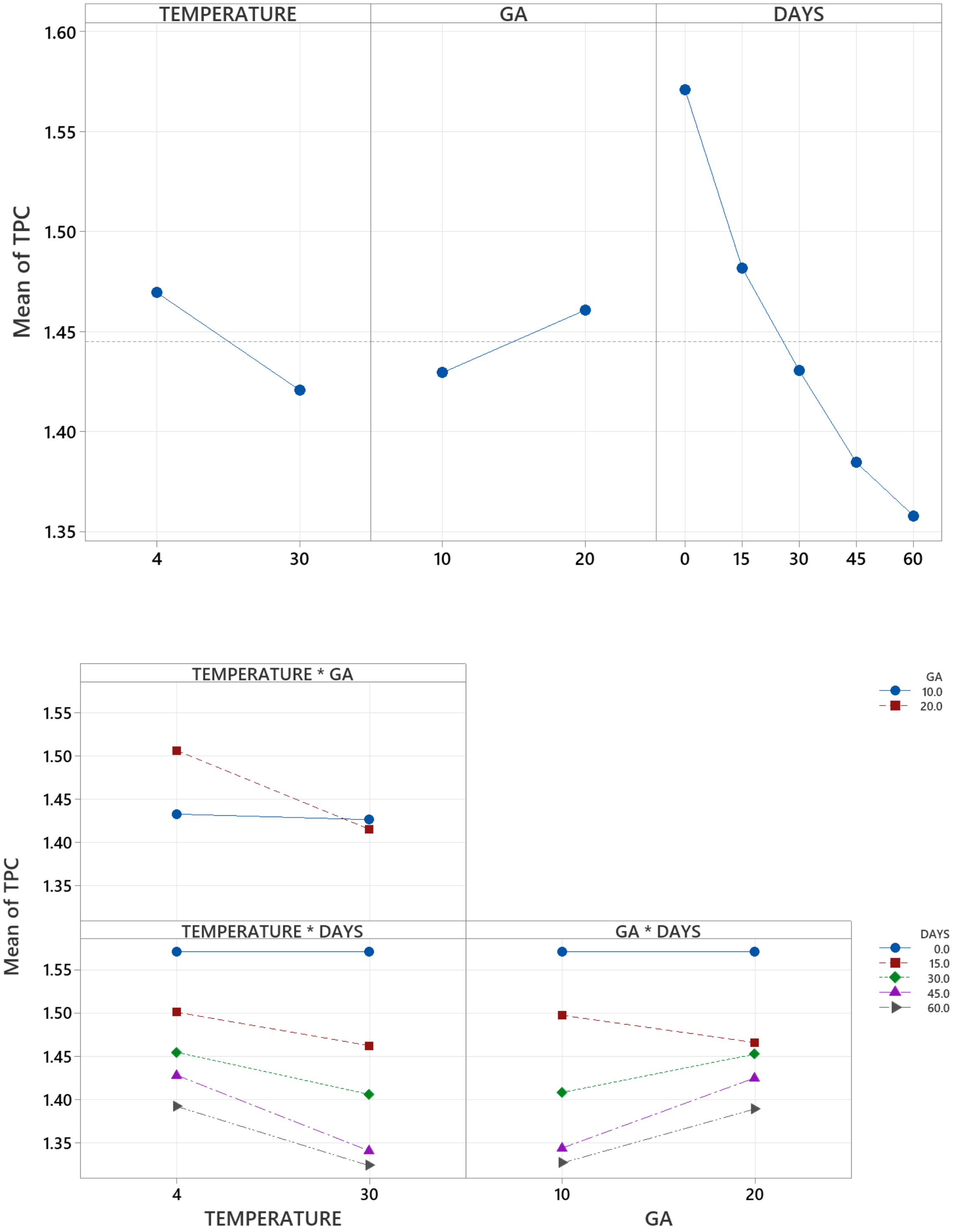

Figure S2. Main effects plot for flavonoids.

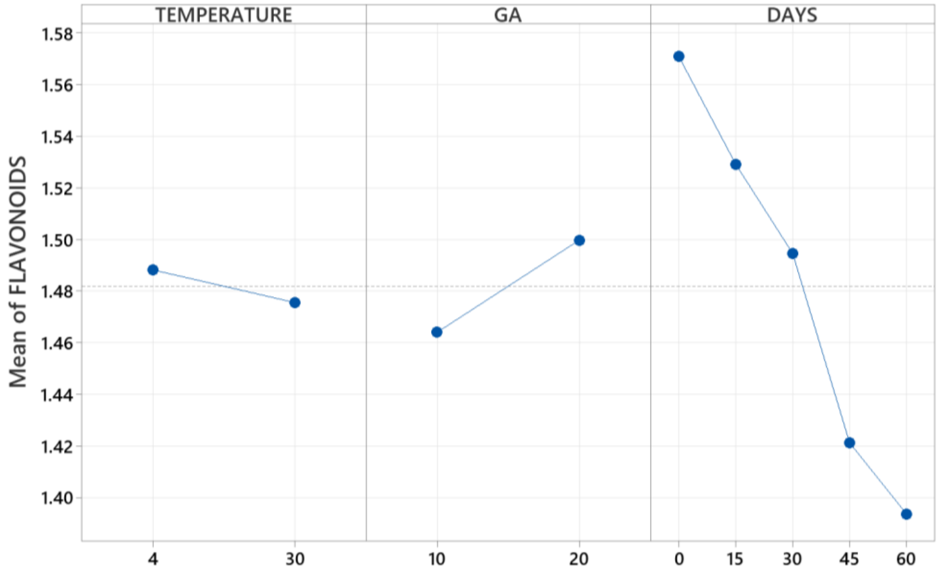

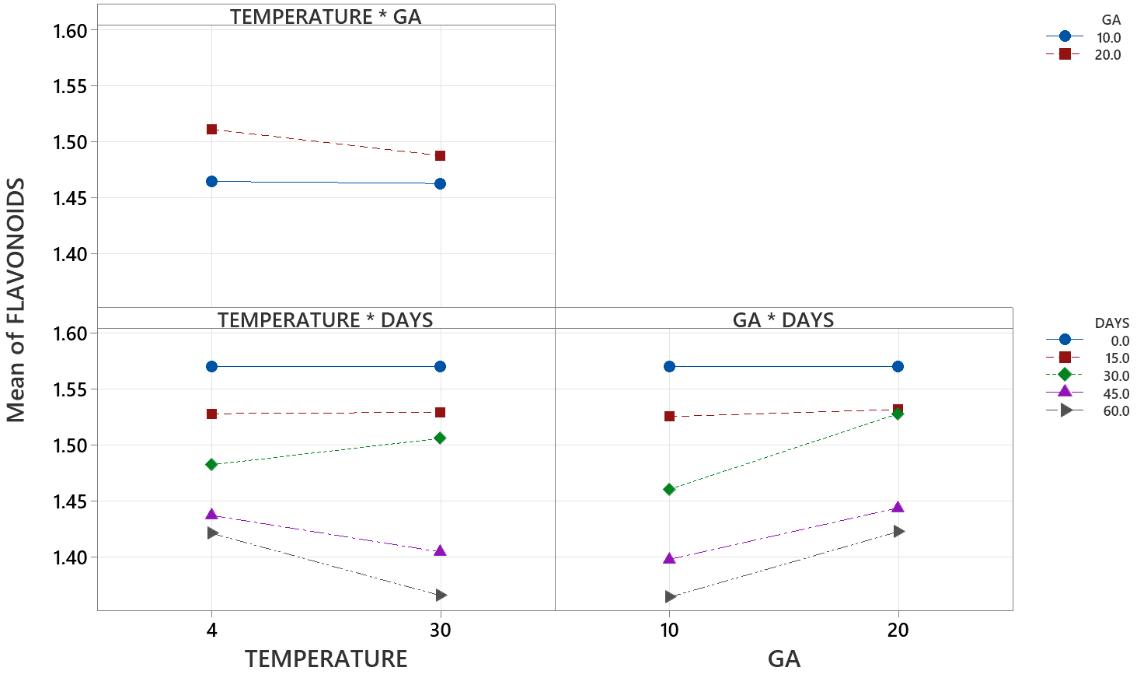

Figure S3. Main effects plot for tannins.

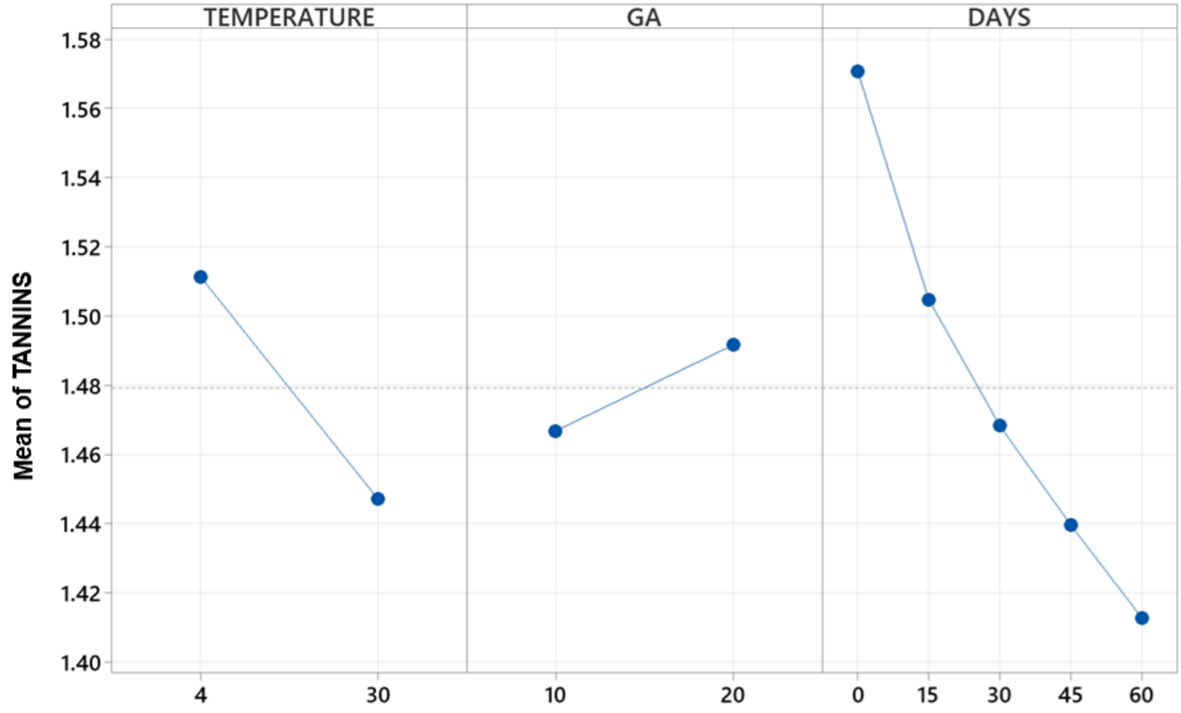

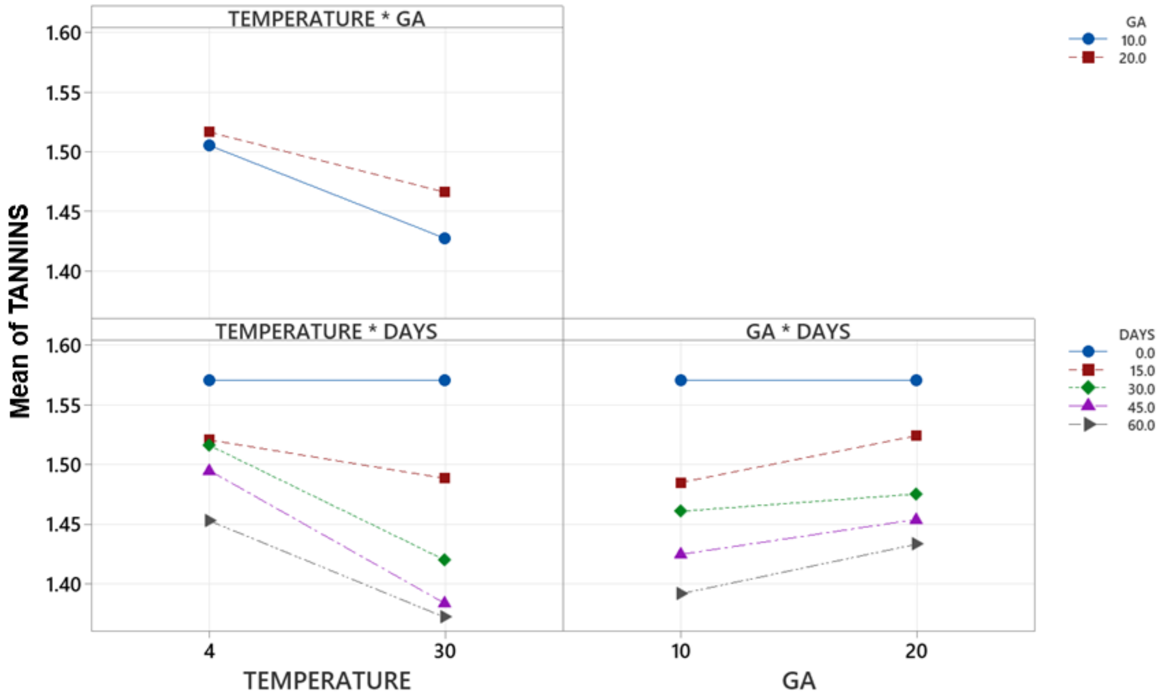

Figure S4. Main effects plot for DPPH.

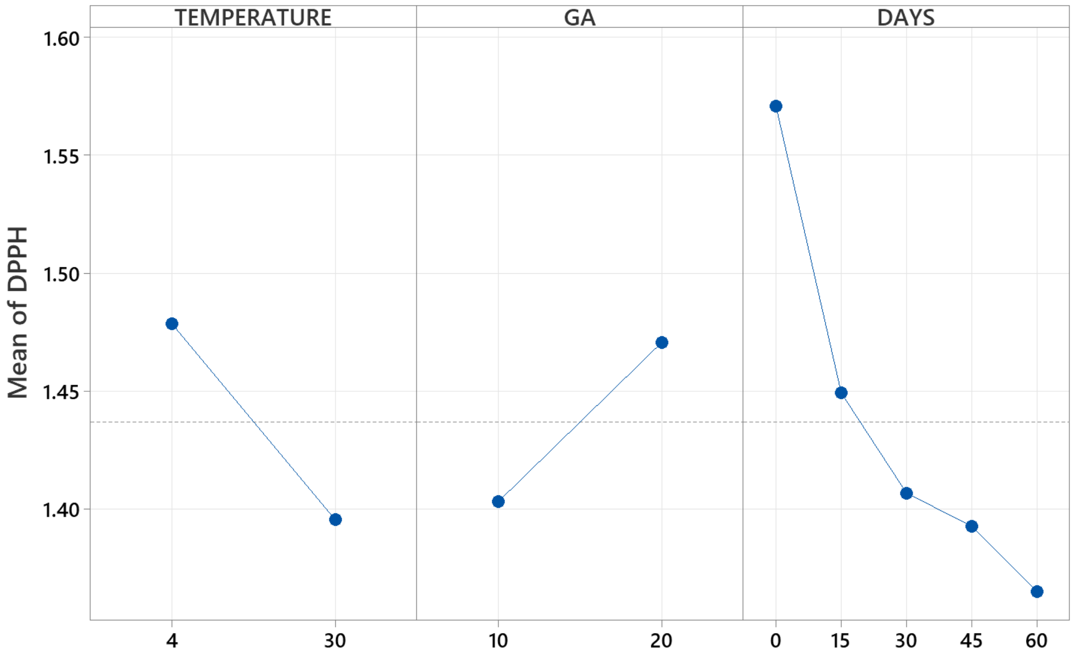

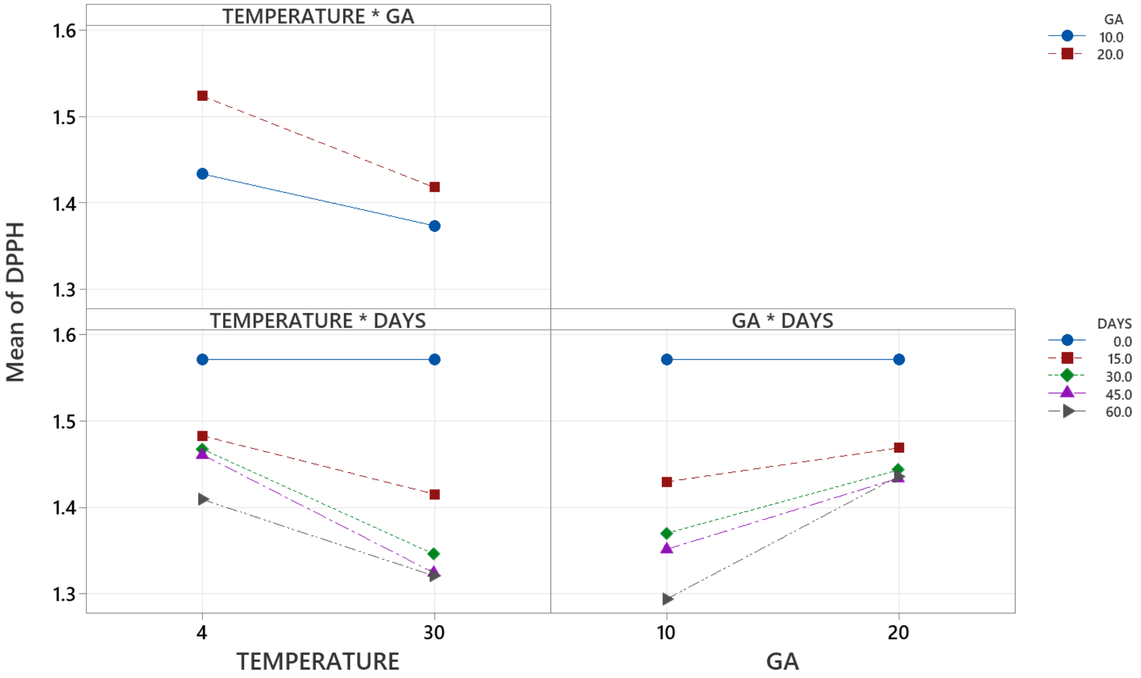

Figure S5. Main effects plot for ABTS.

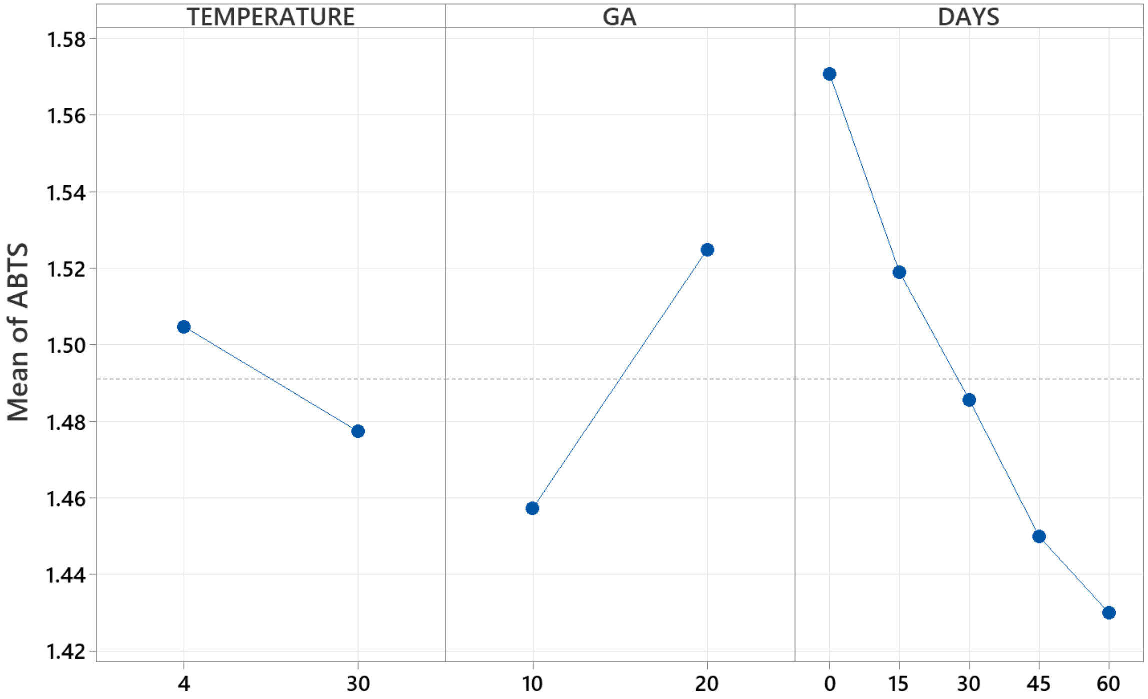

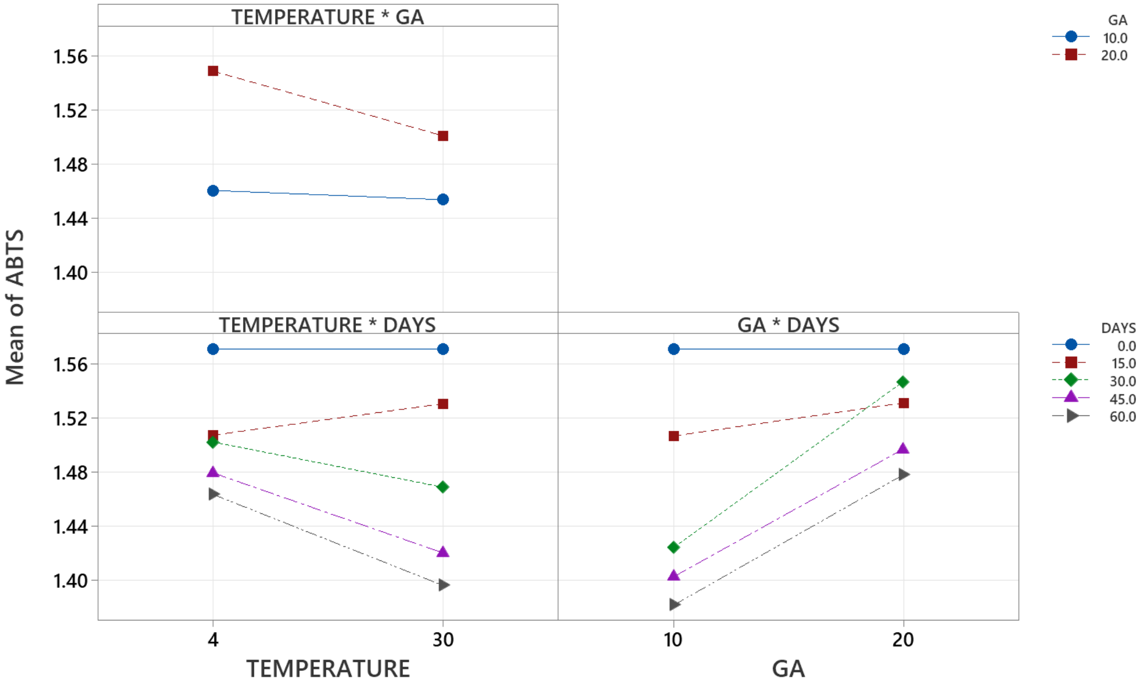

Table S1. Raw storage data.

GA 10%, 4°C						
**Measurement**	**0**	**15**	**30**	**45**	**60**	**% loss**
Phenolics	100.00	95.84	94.70	93.53	92.11	
Phenolics	100.00	102.38	98.60	97.64	98.36	
Average	100.00	99.11	96.65	95.59	93.66	6.34
Deviation standard		4.63	2.75	2.91	4.42	
Flavonoids	100.00	101.28	98.43	97.30	95.74	
Flavonoids	100.00	99.14	97.98	97.02	97.74	
Average	100.00	100.21	98.20	97.16	96.74	3.26
Deviation standard	0.00	1.51	0.32	0.19	1.42	
Tannins	100.00	99.14	100.43	99.49	98.80	
Tannins	100.00	100.88	99.59	98.13	96.96	
Average	100.00	100.01	100.01	98.81	97.88	2.12
Deviation standard	0.00	1.23	0.60	0.96	1.30	
DPPH	100.00	97.24	98.10	97.27	94.81	
DPPH	100.00	98.94	97.15	98.23	92.98	
Average	100.00	98.09	97.63	97.75	93.89	6.11
Deviation standard	0.00	1.20	0.67	0.68	1.30	
ABTS	100.00	97.58	97.97	97.21	96.24	
ABTS	100.00	99.90	98.30	98.37	97.80	
Average	100.00	98.74	98.13	97.79	97.02	2.98
Deviation standard	0.00	1.64	0.24	0.82	1.10	

GA 20%, 4°C						
**Measurement**	**0**	**15**	**30**	**45**	**60**	**% loss**
Phenolics	100.00	99.47	98.73	99.81	97.17	
Phenolics	100.00	101.05	100.07	98.69	98.27	
Average	100.00	100.26	99.40	99.25	97.72	2.28
Deviation standard	0.00	1.12	0.95	0.79	0.78	
Flavonoids	100.00	99.41	99.94	98.20	99.22	
Flavonoids	100.00	100.43	99.65	99.64	97.78	
Average	100.00	99.92	99.80	98.92	98.50	1.50
Deviation standard	0.00	0.72	0.20	1.02	1.02	
Tannins	100.00	100.30	99.66	100.30	99.54	
Tannins	100.00	98.86	99.11	99.13	98.62	
Average	100.00	99.58	99.38	99.72	99.08	0.92
Deviation standard	0.00	1.02	0.39	0.83	0.66	
DPPH	100.00	100.16	100.32	99.68	99.78	
DPPH	100.00	99.34	98.91	99.32	99.00	
Average	100.00	99.75	99.61	99.50	99.39	0.61
Deviation standard	0.00	0.58	1.00	0.25	0.55	
ABTS	100.00	101.69	100.98	100.97	100.55	
ABTS	100.00	99.57	100.06	99.52	99.29	
Average	100.00	100.63	100.52	100.25	99.92	0.08
Deviation standard	0.00	1.50	0.65	1.02	0.89	

GA 10%, 30°C						
**Measurement**	**0**	**15**	**30**	**45**	**60**	**% loss**
Phenolics	100	99.24	98.52	94.55	92.95	
Phenolics	100	100.67	96.90	93.41	91.60	
Average	100	99.95	97.71	93.98	92.27	7.73
Deviation standard	0	1.01	1.15	0.80	0.96	
Flavonoids	100	100.21	99.50	97.29	95.52	
Flavonoids	100	99.25	98.99	96.57	93.72	
Average	100	99.73	99.24	96.93	94.63	5.37
Deviation standard		0.69	0.36	0.51	1.28	
Tannins	100	100.65	98.86	97.10	97.14	
Tannins	100	99.38	98.55	95.98	96.29	
Average	100	100.01	98.70	96.54	96.71	
Deviation standard		0.89	0.22	0.80	0.60	3.29
DPPH	100	97.37	94.67	92.54	90.30	
DPPH	100	98.27	93.29	91.25	91.72	
Average	100	97.82	93.98	91.89	91.01	8.99
Deviation standard		0.64	0.98	0.91	1.00	
ABTS	100	100.33	97.63	96.97	95.24	
ABTS	100	99.53	97.52	95.98	96.35	
Average	100	99.93	97.58	96.48	95.80	4.20
Deviation standard		0.56	0.08	0.70	0.78	

GA 20%, 30°C						
**Measurement**	**0**	**15**	**30**	**45**	**60**	**% loss**
Phenolics	100.00	96.97	96.03	95.75	95.27	
Phenolics	100.00	97.10	97.49	95.37	95.82	
Average	100.00	97.04	96.76	95.56	95.54	4.46
Deviation standard	0.00	0.09	1.03	0.27	0.39	
Flavonoids	100.00	100.60	100.64	98.20	96.34	
Flavonoids	100.00	99.41	99.26	96.90	97.45	
Average	100.00	100.00	99.95	97.55	96.89	3.11
Deviation standard	0.00	0.84	0.97	0.92	0.78	
Tannins	100.00	97.37	96.32	95.99	94.69	
Tannins	100.00	99.25	96.74	97.03	96.13	
Average	100.00	98.31	96.53	96.51	95.41	4.59
DESV	0.00	1.33	0.29	0.74	1.02	
DPPH	100.00	97.44	96.43	96.53	95.55	
DPPH	100.00	97.28	95.47	95.18	96.84	
Average	100.00	97.36	95.95	95.86	96.19	3.81
Deviation standard	0.00	0.11	0.68	0.95	0.91	
ABTS	100.00	100.846	100.403	99.0918	98.6067	
ABTS	100.00	99.1523	99.0999	98.3255	97.2161	
Average	100.00	100.00	99.75	98.71	97.91	2.09
Deviation standard	0.00	1.20	0.92	0.54	0.98	
